# Review of Temporary Crating of Farrowing and Lactating Sows

**DOI:** 10.3389/fvets.2022.811810

**Published:** 2022-03-17

**Authors:** Sébastien Goumon, Gudrun Illmann, Vivi A. Moustsen, Emma M. Baxter, Sandra A. Edwards

**Affiliations:** ^1^Animal Physiology, Institute of Agricultural Sciences, ETH Zürich, Zürich, Switzerland; ^2^Department of Ethology, Institute of Animal Science, Prague, Czechia; ^3^Faculty of Agrobiology, Food and Natural Resources, Czech University of Life Sciences Prague, Prague, Czechia; ^4^SEGES Danish Pig Research Centre, Copenhagen, Denmark; ^5^Animal Behaviour and Welfare, Animal and Veterinary Sciences Group, Scotland's Rural College, Edinburgh, United Kingdom; ^6^School of Natural and Environmental Sciences, Newcastle University, Newcastle upon Tyne, United Kingdom

**Keywords:** temporary crating, free farrowing, permanent crating, lactation, sows, piglets, welfare

## Abstract

Temporary crating (TC) provides lactating sows with the opportunity to move more freely after crate opening a few days after parturition. The aim of this paper was to evaluate whether TC gives overall welfare improvement when compared to permanent crating or free farrowing. This review shows that when pens with TC allow the sows to turn during the majority of time in the farrowing unit, it is the pen design and period of confinement in a crate within it that influence the extent to which different functional and motivated behaviors can be fulfilled. This review also indicates that there are at least short-term benefits to sows when confinement is reduced, as shown by reported increases in motivated behaviors such as exploration and interactions with piglets when not permanently crated. It remains unclear whether there are any longer-term beneficial effects (until or beyond weaning) due to the paucity of studies. Furthermore, it is uncertain whether the observed short-term benefits translate to other welfare indicators. Research findings indicate no reduction in the frequency of stereotypies or body lesions and do not provide a clear answer regarding sow stress response when released from confinement. Compared to free farrowing, TC appears beneficial for reducing piglet mortality. The impact of the time of onset of TC on the farrowing process and piglet mortality have been inconsistent. While confinement before farrowing prevents nest building behavior, consequences of this for sow physiology have been ambiguous. Confining the sow briefly after farrowing may be the best compromise, allowing the sow to perform motivated nest-building behavior, but the risks of crushing during the unconfined farrowing period may increase. Subsequent crate reopening seems to increase piglet mortality but only if done earlier than 3–5 days after farrowing. The review also provides methodological considerations, a proposal for consistent and accurate terminology when describing systems and highlights gaps of knowledge. In conclusion, TC is a step forward to better pig welfare compared to the farrowing crate, as it allows some freedom of movement for sows without impairing piglet welfare. However, more comprehensive research is needed to draw sound conclusions as to whether TC is a viable transition from permanent crating to free farrowing.

## Introduction

In global pig production, conventional housing for farrowing and lactating sows continues to be in farrowing crates, which are close confinement systems that limit the sow's movements to standing and lying but without the possibility to turn. The farrowing crate was first introduced in the 1960s to reduce piglet mortality as a result of overlying by the sow and to allow more targeted interventions by stockworkers. An additional economic advantage of such a system is that the sow and litter are kept on a minimal spatial footprint [~1.23 m^2^ crate within 3.6–4.6 m^2^ pen—(1)], typically on fully or partially slatted floors above slurry pits and thus requiring minimal labor for maintaining hygiene. A notable advantage of the crate is more control around the farrowing period, including a reduction in crushing of piglets by the sow. However, higher mortality as a result of other causes such as stillbirth or starvation has been reported in some (2–4) but not all studies [e.g., (5)].

The welfare issues for crated sows are well documented [for reviews see (1, 6)], and there continues to be public pressure to ban their use. Legislation already restricts use in Sweden, Switzerland, and Norway, while New Zealand, Austria, and Germany have announced phasing out of farrowing crates by 2025, 2033, and 2036, respectively. Pressure to abolish systems involving close confinement is apparent in the rest of Europe, with a recent petition to the European Citizen Initiative (ECI) by the “End the Cage Age” campaign attracting over 1.4 million signatories from more than 18 member states with support from over 170 organizations, thus meeting the criteria to “invite the Commission to submit a proposal for a legal act to implement the EU Treaties.” In response, the Commission said that “by the end of 2023, a legislative proposal to phase out, and finally prohibit the use of cage systems for all animals mentioned in the Initiative” will be put forward. Outside Europe, consumer surveys in Brazil suggest that citizens have a negative attitude toward farrowing crates and consider that reducing piglet mortality does not fully justify their continued use (7).

Piglet mortality, however, is a continuing concern for producers, and the risk of increased mortality in free farrowing systems is a major barrier to uptake of alternatives. Successful adoption and consistent performance have been reported in countries operating non-crate systems for a long time; a recent report from Switzerland documented performance from over 330,000 litters from 255 free farrowing farms. They compared 2017 figures with those from 2003, when farmers were in the transition period from crates to loose housing, and report no significant difference in mortality despite an increase in litter size (8). Elsewhere, there are mixed reports of success. Sweden, who has had a crate ban since 1987, has seen piglet mortality levels rise in the last 10 years. This is partly a result of increasing litter size (9), although national herd data from several countries suggests that Sweden's mortality level compared to litter size is an outlier, lying above the regression line for crates (10). Large litters have a known association with piglet mortality, and a recent Swedish study comparing performance in temporary confinement and loose systems highlighted that, regardless of farrowing system, more piglets died in large litters compared to small ones (11). The consistent production of supernumerary piglets (i.e., piglets in excess of the number of functional teats) requires significant interventions by staff to promote survival. This is perhaps one of the major barriers to adoption of a truly free farrowing system (i.e., zero confinement throughout farrowing and lactation), along with concerns about costs, not only of installation but also for long-term production. Producers want to retain the advantages of the crate in controlling sow movement (to reduce crushing), allowing localized heating at the birth site and facilitating safe targeted interventions by staff to promote piglet survival such as assisted suckling, split suckling, and cross-fostering. All of these factors have a greater necessity as a result of an increased prevalence of very large litter sizes and explain why systems that permit the use of a crate temporarily are becoming more popular and why Austria and Germany are permitting “crating during the critical period for piglet survival” within their legislation (4^1^ and 5^2^ days maximum, respectively). Within Denmark's pledge to have 10% of its herd farrowing in alternatives by 2021, it will also permit the temporary use of crates (12).

The sow welfare concerns regarding use of traditional crates, yet the desire to retain a confinement option to promote piglet survival, have led to the evolution of both systems with the option of a crate being utilized temporarily within a pen, and an expectation that the sow will be loose for the majority of the time, or conventional crate systems with the option to simply open the crate, with the expectation the sow will be let loose at some point during lactation (see Section Pen Design Variability: From Crate to Temporary Confinement or From Loose Housing to Temporary Confinement? for Further Discussion). Quantifying the current use of TC systems in different countries is difficult. In Denmark, it is estimated that ~4% of their breeding herd use TC, with a few producers operating completely free farrowing (13). In Austria, there are reports of 5% having started the transition phase to TC or free farrowing, which must be in place by 2033 (14). As a proxy for determining global interest in these systems, demographics on published research on “free” farrowing systems in the last 20 years shows that activity is not restricted to Europe. New Zealand, Australia, and the USA, and some Asian countries, all have active research programs. These are dominated by trialing of TC systems of varying designs and with different confinement periods.

This review aims to critically evaluate the existing literature to determine if there is robust evidence regarding whether: (i) TC gives overall welfare improvement when compared to permanent crating, and (ii) TC gives overall welfare improvement when compared to free farrowing. Improvements will be examined from the perspectives of both the sow and her piglets, using behavioral, physiological, and performance data where available. Critical evaluation of the evidence will allow identification of knowledge gaps and proposals for the direction of future studies. These insights will assist various stakeholders in making decisions about whether TC systems offer a viable transition from permanent crating to free farrowing.

## Methodology

### Terminology

Various terms are used to describe different maternity systems and practices that represent alternatives to the conventional farrowing crate. The most common umbrella term is “free farrowing,” which should indicate zero confinement. However, in many cases, sows are actually crated and not able to freely turnaround during parturition. The synonymous use of the terms “crating” and “confinement” is also common, but we have and will (see Section Pen Design Variability: From Crate to Temporary Confinement or From Loose Housing to Temporary Confinement?) argue that the distinction is important. Table 1 defines the terminology we will use throughout this review. We propose that these terms (with these definitions) be adopted for future investigations and discussions in this field, which will aid clarity in future attempts to evaluate particular systems or practices. The capital letter D followed by a number will be used to refer to the number of days after farrowing, the latter being D0 or D1 depending on the definition used in research papers.

**Table 1:** Definitions of indoor farrowing and lactation systems, practices, and terms.

**Term**	**Synonyms**	**Definition**
Free farrowing	Zero confinement	A system or practice where there is no possibility to confine the sow in a crate when in farrowing and lactation accommodation. Sows are able to freely turn around at all times Pens may have feeding stalls or areas to separate the sow from the piglets temporarily for management purposes, but the fixtures would not permit crating for farrowing
Temporary crating	Temporary confinement Loose lactation Free lactation	A system or practice where the sow is confined in a crate (without the possibility to turn around) for a certain period of time, but not the whole of lactation
Permanent crating	Farrowing crates	Conventional system or practice where the sow is permanently confined in a farrowing crate from entry into the maternity accommodation until weaning

### Literature Selection

Literature searches were not restricted to any specific database and included Web of Science and Google Scholar. There was no specific language criterion, although the majority of peer-reviewed papers were in English (~85%). Terms used for searches were different combinations of the keywords [Temporary AND (crate OR confinement)] AND (Farrowing OR lactation) AND (Pig OR Sow OR Sus Scrofa). After screening titles and abstracts for relevant research on TC, the review was then supplemented with other relevant references cited in these publications or known to the authors. In total, 33 research papers and 1 scientific report have been considered in this review to compare temporary crating systems to free farrowing (eight papers + report) and permanent crating systems (all papers + report).

### Evaluation

This review describes the evolution of farrowing pen design and the associated challenges and discusses gaps in the literature regarding the evidence for legislative decisions on aspects of housing (e.g., space). Management decisions such as when to confine pre-farrowing and when to release post-farrowing are discussed, and the positive and negative impacts on sow and piglet behavior, welfare, and performance are addressed.

## Pen Design Variability: From Crate to Temporary Confinement or From Loose Housing to Temporary Confinement?

One of the factors that makes objective evaluation of TC systems difficult is the great variety in the pen designs that have been used (see Table 2). There are differences in the nature of the crate, the size and flooring of the pen, and the equipment used to ensure the thermal comfort of piglets. The extent to which each of these components might be considered optimal, or even adequate, can be questioned in some studies. To accommodate the sow's opportunity to turn around when loose (15), most pens for loose sows are larger (4–8 m^2^) (Table 2) than the space provided in conventional farrowing crate pens [~1.23 m^2^ crate within 3.5–4.5 m^2^ pen; (18, 27, 46)]. As a result, farmers need to either build new facilities or reduce the number of pens, where the latter includes reduction in herd size, both involving long-term cost implications. Even though farrowing and lactation pens basically only need places for sows to eat, drink, rest, and dung and a creep area for piglets, the differences in dimensions of sows and piglets, the increasing litter sizes, the work conditions for caretakers, and differences in construction lead to a large variation in the pens available and used both in trials (32, 37) and on farms (19, 28, 38, 39). The higher neonatal piglet mortality observed in studies where sows were loose during farrowing compared to sows that were in crates (5, 24, 54) has led to consideration of the opportunity to temporarily confine the sows in pens designed for loose sows (26, 28, 32). However, some pens with TC used in trials and available on the market have more similarities with traditional farrowing crates for confined sows than with designed pens for loose sows. Their starting point is a typical farrowing crate that opens up after a certain period post-farrowing. These might be called swing-side crates or modified crates and are generally built on the similar spatial footprint as conventional crates, with fully or partially slatted floors. However, it can be argued that even conventional crates and the minimal spatial footprint they occupy are no longer suitable for modern hyperprolific sows and litters, both being notably larger than when crates were first introduced (55, 56).

**Table 2:** Pen dimensions^a^ and area (total, solid floor, accessible for sows, only accessible for piglets) and presence of covered creep area.

**References**	**Commercial name and/or named used in paper for TC**	**Pen dimensions and area**	**Area with solid floor**	**Area accessible for sow**	**Area accessible for piglets**	**Covered creep area**	**Dimensions TC**
Caille et al. (16)	Partial freedom	2.6 × 2.25 = 5.85 m^2^	Fully slatted or fully solid floor	4.8 m^2^	1 m^2^	Not specified	0.60 × 2.40 = 1.44 m^2^
Ceballos et al. (17)	Hinged farrowing crate	2.0 × 2.1 = 4.2 m^2^ Perforated plastic flooring	Fully slatted flooring Only heat pad piglets 0.8 × 0.6 = 0.48 m^2^	3.03 m^2^	1.1 m^2^	Not specified	1.73 × 0.64 = 1.1 m^2^
Ceballos et al. (18)	Hinged farrowing crate	2.36 × 1.7 = 4.0 m^2^ Perforated plastic flooring	Not specified	2.8 m^2^	1.26 m^2^	Yes	2.13 × 0.63 = 1.35m^2^
Chidgey et al. (19–22)	Combi.Flex (Vissing Agro)	2.25 × 2.6 = 5.85 m^2^	Fully slatted flooring Only heat pad piglets	5 m^2^	0.84 m^2^	Yes	2.1 × 0.6 = 1.26 m^2^ (adjustable)
Choi et al. (23)	Openable pens	1.9 ×1.4 = 2.66 m^2^	Not specified	1.9 m^2^	1.4 m^2^	Not specified	1.9 ×0.6 = 1.23 m^2^
Condous et al. (24)	Swing-sided	2.8 ×2.15 = 6.0 m^2^	Fully slatted plastic tiles	5 m^2^	App 0.96 m^2^	Yes	1.43–2.07 m^2^
Farmer et al. (25)	Modified farrowing pen	(2.1 + 1.6) ×1.5 = 5.55 m^2^	1.5 ×1.6 = 2.4m^2^	3.6 m^2^	1.89 m^2^	No	2.1 ×0.6 = 1.26 m^2^
Goumon et al. (26)	Farrowing pen equipped with movable bars	(0.50 + 0.65 + 1.20) ×2.5 = 5.88 m^2^	5.88 m^2^	4.63 m^2^	1.25 m^2^	No	2.5 ×0.65 = 1.62 m^2^
Hales et al. (27)	Swing side crate	1.75 ×3.0 = 5.25 m^2^	2.8 m^2^	Not specified	Not specified	Yes	1.70 ×0.55 (front) or 1.70 ×0.62 (back) m = 0.93–1.05 m^2^
Hales et al. (28, 29)	SWAP	2.10 ×3.0 = 6.3 m^2^	3.78 m^2^	5.3 m^2^	1 m^2^	Yes	2.35 ×0.8 = 1.88 m^2^
Hansen (30)	Ten designs^b^	2.0–2.7 ×2.4–3.3 = 5.0–6.9 m^2^	0.7–2.9 m^2^	Not specified	0.7–1.0 m^2^	Yes/No	177–240 ×0.52–0.86 m
Hansen et al. (31)	SWAP	2.10 ×3.0 = 6.3 m^2^	3.78 m^2^	5.3 m2	1 m^2^	Yes	2.35 ×0.8 = 1.88 m^2^
Heidinger et al. (32)	Five designs^c^	5.5–7.4 m^2^	Not specified in summary text	Not specified in summary text	Not specified in summary text	Yes/No	Not specified in summary text
Höbel et al. (33)	Petra and Freya	5.5–6.9 m^2^	Not specified in summary text	Not specified in summary text	Not specified in summary text	Yes/No	Not specified in summary text
Illmann et al. (34)	Farrowing pen equipped with movable bars	(0.50 + 0.65 + 1.20) ×2.5 = 5.88 m^2^	5.88 m^2^	4.63 m^2^	1.25 m^2^	No	2.5 ×0.65 = 1.62 m^2^
Illmann et al. (35)	Farrowing pen equipped with movable bars	(0.50 + 0.65 + 1.20) ×2.5 = 5.88 m^2^	5.88 m^2^	4.63 m^2^	1.25 m^2^	No	2.5 ×0.65 = 1.62 m^2^
Kinane et al. (36)	Free lactation pen	2.12 ×2.61 = 5.5 m^2^	Only heat pad	3.4 m^2^	2.1 m^2^	No	3.4 m^2^
King et al. (37–39)	Straw-based pens	Indoor: 1.9 ×2.3 = 4.37 m^2^ + outdoor 1.9 ×2.55 = 4.85 m^2^	4.37 + 4.85 = 9.22 m^2^	7.6 m^2^	1.61 m^2^	Yes	Not confined
King et al. (37–39)	360	1.8 ×2.5 = 4.5 m^2^	Only heat pad 1.2 ×0.4 = 0.48 m^2^	2.25 m^2^ (4 m^2^ at shoulder height)	2.3 m^2^	No	2.5 ×0.9 = 2.25 m^2^
Lambertz et al. (40)	Loose-housing pens	1.85 ×2.5 = 4.5 m^2^	Fully slatted Floor heated piglet area (dimensions not specified)	2.8 m^2^	1.7 m^2^	No	From 0.55 to 0.70 ×2.0 = 1.1–1.4 m^2^
Loftus et al. (41)	Freedom Farrowing pen	2.0 ×2.8 = 5.6 m^2^	Fully slatted except creep area (dimensions not specified)	3.2 m^2^	2.4 m^2^	No	0.57 ×2.0 = 1.14 m^2^
Lohmeier et al. (42)	Farrowing pen1 and Farrowing pen2	7.0–7.6 m^2^	Not specified	3.2–4.3 m^2^	3.8–3.3 m^2^	Yes	Not specified
Mack et al. (43)	Open crate	2.0 ×2.1 = 4.2 m^2^	Only heatpad	2.9 m^2^	1.3 m^2^	No	0.64 ×1.73 = 1.11 m^2^
Maschat et al. (44)	Five designs^3^	5.5–7.4 m^2^	Not specified in summary text	Not specified in summary text	Not specified in summary text	Yes/No	Not specified in summary text
Morgan et al. (45)	Modifications of farrowing crate	2.05 ×2.92 = 5.99 m^2^	Fully slatted covered with rubber mat for piglets (creep area)	Not specified	Not specified	No	Width not specified; length = 1.8 m
Moustsen et al. (46)	Combi-farrowing pen	1.8 ×2.6 = 4.68 m^2^	Solid in creep area, drained at feeder, slatted away from feeder	Not specified	Not specified	Yes	Not specified
Nowland et al. (47)	360	1.8 x 2.5 = 4.5 m^2^	Only heat pad 1.2 ×0.4 = 0.48 m^2^	2.25 m^2^ (4 m^2^ at shoulder height)	2.3 m^2^	No	2.5 ×0.9 = 2.25 m^2^
Olsson et al. (11)	Temporarily confinement	3.35 ×1.95 = 6.5 m^2^	1.75 ×1.95 = 3.4 m^2^	Not specified	1 m^2^	Yes	Not specified
Oostindjer et al. (48)	Loose housing	Barren pens: 9.2 m^2^ or enriched pens: 18.4 m^2^ (dimensions were not specified)	Barren pens: 65% solid /35% slatted. Enriched pens: 65% covered with wood shaving and 35% covered in peat	While crated: 1.68 m^2^; Loose barren pen: 7.15 m^2^; Loose enriched pen: 16.38 m^2^	Not specified	No	1.68 m^2^
Pedersen et al. (49)	Swing side crate	2.46 ×1.78 = 4.38 m^2^	Only in creep area	Not specified	Not specified	Yes	Not specified
Salaün et al. (50)	Partial freedom	2.6 ×2.25 = 5.85 m^2^	Fully slatted or fully solid floor	4.8 m^2^	1 m^2^	Not specified	0.60 ×2.40 = 1.44 m^2^
Singh et al. (51)	Lactation pen	Crate-pen: 1.5 ×2.0 = 3 m^2^ Loose pen Model 1: 1.8 ×2.5 = 4.5 m^2^ Model 2: 1.7 ×2.4 = 4.1 m^2^	Only creep (1.23 ×0.45 = 0.55 m^2^) Otherwise fully slatted plastic flooring	Model 1: 3.95 m^2^ Model 2: 3.55 m^2^	0.55 m^2^	No	0.6 ×2.0 = 1.2 m^2^
Spindler et al. (52)	Openable crate	5.35–5.75 m^2^	Fully slatted Only mat in creep area	2.66 m^2^	0.75 m^2^	Yes	Not specified
Verhovsek et al. (53)	Trapez pen	6.7 m^2^	Partially slatted floor. Dimensions not specified	Not specified	Not specified	Not specified	Not specified

*^a^Measurements are listed as reported in papers.*

^b^*Ten pen types named Free Move, BeFree, Welsafe, Opti Farrow, ProDromi, Well Fair Pen, Wing, 360, SWAP, and JLF-14, respectively*.

^c^*Five pen types named Flügelbucht, Knickbucht, Trapezbucht, SWAP, and Pro Dromi, respectively*.

Where the sows feed, drink, stand, and lie and where they defecate and urinate, they need at minimum their own length. Moustsen et al. (55) published the physical dimensions of 322 Danish crossbred sows (measured in 2004), and in 2018, Nielsen et al. published the physical dimensions of 405 Danish crossbred sows (measured in 2017). In Nielsen et al. (56), the mean depth, width, length, and height were estimated to be 66, 43, 192, and 90 cm, respectively, and for the 95 percentile, it was 72, 48, 203, and 98 cm for the full-grown sows (parity ≥5). Accordingly, sows' body dimensions were found not to have increased further since 2004 (55, 56). For standing up and lying down, the sows need their own length plus 50 cm and their own width plus 40 cm (57). In contrast to a farrowing crate, which is designed for the sow not to be able to turn around, pens for a free farrowing sow and for temporary confinement must allow space for the sows to turn around. The area needed depends on sow dimensions. Using an allometric approach, Baxter et al. (15) suggested that a planar width of 153 cm and a planar area of 3.17 m^2^ were necessary to allow unobstructed turning for sows with the 95-percentile weight. Given the variable size and body conformation of different modern sow genotypes (55, 56), this estimate requires empirical validation.

For unimpeded nest building, the sows should be loose before farrowing, and preferably the pens should have a solid floor of sufficient size for the sows to turn around and nest build (15, 58). In addition, to obtain a high level of hygiene, the sows should have access to, and be motivated to use, a slatted area sufficient in size to achieve a separated excretory area (59). The need to increase pen space has been recognized within national legislation: Sweden^3^ and Switzerland^4^ have similar language about sows being kept loose after giving birth and being able to move and turn in the space without difficulty, with further stipulations about minimum space allowances [respectively 6 m^2^ (with a minimum lying area of 4 m^2^ for the sow) and 5.5 m^2^ (with 2.25 m^2^ allocated to the sow lying area)]. Austria and Germany specify minimum pen sizes of 5.5 and 6.5 m^2^, respectively, with further language about sows being able to turn freely (when unconfined).

The space allocated for piglets is also highly variable between studies (60). In 2004, Moustsen and Poulsen published dimensions of 109 piglets, and in 2017, Moustsen and Nielsen, published dimensions of 202 piglets. When piglets are lying in semi-recumbent posture, they take up ~0.1 m^2^ per piglet at 4 weeks of age, with a height of 21–29 cm, length of 36–53 cm, and shoulder width of 8–13 cm (61). One challenge is free or easy access to the teats while the sows are confined, which requires that the inner width of the confinement is wider than the depth (from the backbone of the sow to the base of the teats) of the sows. Furthermore, the distance between the teats and the pen wall needs to accommodate the length of piglets when suckling. Although it is not specified, it is expected that the measurements reported in publications regarding width of the farrowing crate are inside dimensions and so available for sow and piglets when nursing while the sow is confined. In the papers, which have specified the dimensions, the measurements range from 52 cm (30) to 90 cm (37, 47), where 52 cm is insufficient for at least full-grown Danish crossbred sows (56). When the sows are let loose, features in pens such as the “wings” or similar structures used to confine sows, and the positions they are “mounted” in when the sows are loose, influence not only the space available for sows and possible planar width (i.e., width at sow's shoulder height) but also the safety zones for piglets.

The pen designs, both without and with options to confine, referred to in most peer-reviewed publications listed in the review consist of compromises [e.g., (18, 27)], and few, if any, meet all criteria to satisfy the needs of sows, piglets, and staff. In addition, not all pen details are described in peer-reviewed papers [e.g., (27, 32, 44)]. This makes it difficult to compare the pens and to recommend which pen to use in future trials or farm installation or in assisting other stakeholders such as policy-influencers and policymakers. According to the information available (Table 2), the pens differ in total footprint from 2.66 m^2^ (23) to 7.4 m^2^ (32) and 9.22 m^2^ in a pen with access to an outdoor area (37). The area available for sows when loose differs from 1.9 m^2^ (23), 5.3 m^2^ (27), to 7.6 m^2^ in a pen with access to an outdoor area (37) and the available space for piglets protected from the sow varies from 0.44 m^2^ (62) to 2.25 m^2^ (37). However, as mentioned above, it is not only a question of area but also dimensions to accommodate the length, width, height, and locomotion of sows or piglets.

In some designs, there has been a focus on zonation to meet the behavioral predisposition of sows to avoid resting, eating, drinking, and defecating at the same location (15, 63). Some designs supply a part-solid floor for the sows to nest build (27, 32, 37). Zonation is supported by the shape of the pen, where rectangular pens with an excreting area at one short end of the pen have been reported to facilitate the partition of the pen into a dunging area and a lying area by ensuring a larger distance between them (59). Some designs specify separate climatic conditions for sows and piglets by supplying a covered creep area (19, 27). In most of the pens, the creep area had floor heating and/or a heat lamp but was not covered (17, 25, 38), making it difficult to ensure the difference in climate required by sows and piglets (64).

**Figure 1:**
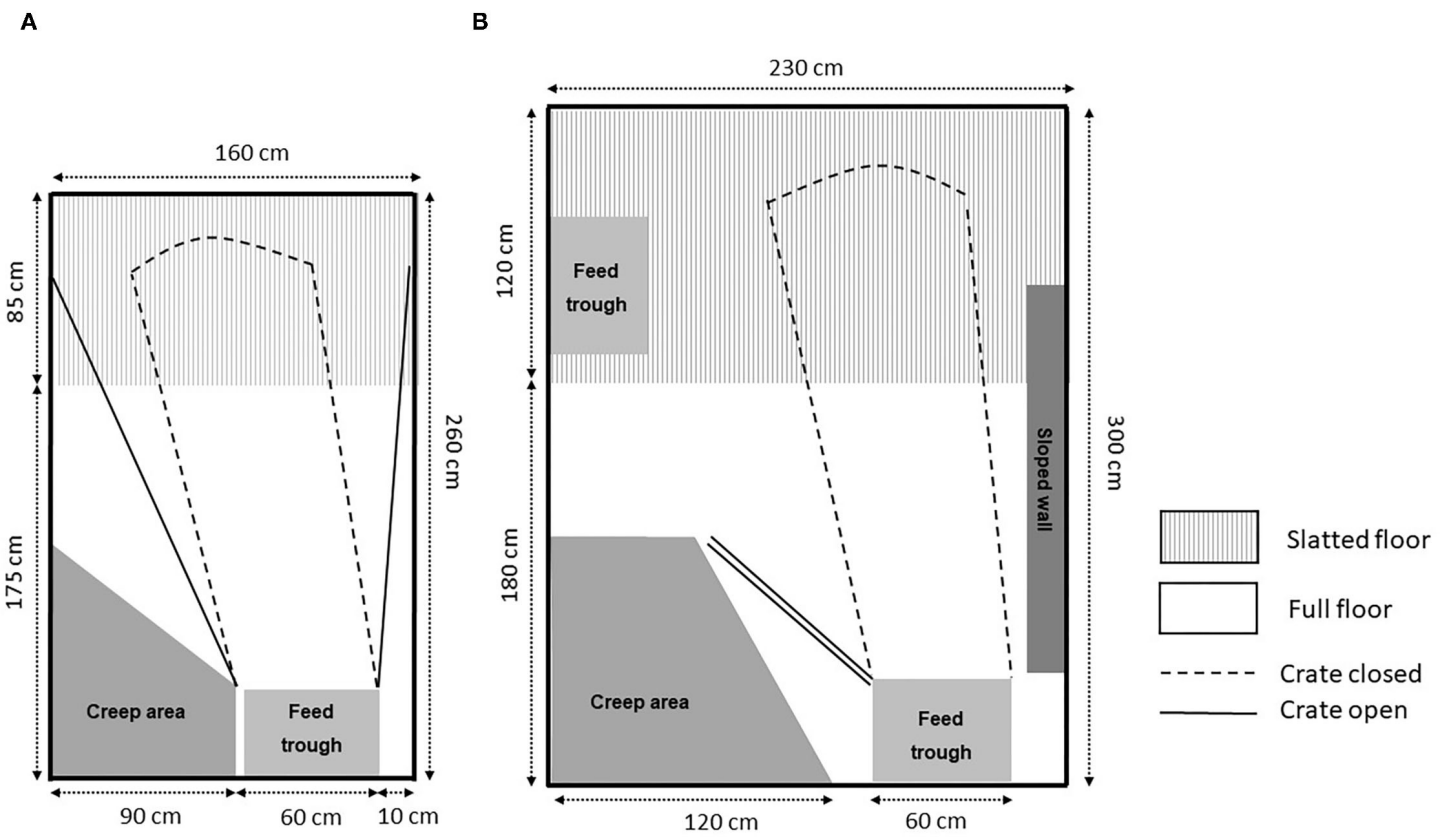
Examples of pens with temporary crating: **(A)** swing-side crate and **(B)** SWAP pen.

Overall, there are pens with a focus in the design on the loose sow and the litter and where temporary confinement is an add-on with the aim of reducing the risk of neonatal piglet crushing (26, 27) (Figure 1). Other pens have more similarities with traditional farrowing crates (45–47) (Figure 1), with limited innovations to meet the sow and piglet behavioral needs (47). The main difference in these limited systems compared with traditional crates is that restrictive bars can be removed, making it easier for piglets to have access to the udder (49) and allowing the sow more room for movement. By shifting the design mindset toward adding a confinement option within a loose pen, rather than partial removal of a crate structure in a crated system, more consideration (in some designs) has been given on how a sow moves dynamically in that confinement space around the critical time window for piglet survival and how the pen could function when she is loose.

## Management Choices in Temporary Crating Systems

In addition to the diversity of housing designs associated with TC, there are also different ways of managing this system. When a TC system is adopted, the most important management decisions relate to the choice of timing for imposing confinement once the sow is present in the pen and then subsequently opening the crate again to give the sow access to the larger pen area.

### The Timing of Confinement

The choice of confinement onset time involves a number of considerations. Any acute stress response associated with this event might adversely impact the farrowing process. Once confined in the crate, the behaviors associated with nest building are constrained, but while the sow is unconfined, measures designed to improve piglet survival are more difficult to implement. These include protection from crushing by restricting the movements of the sow, provision of targeted supplementary heating in areas that benefit neonatal thermoregulation, and safe conditions for stockpeople to intervene and assist piglets failing to thrive.

#### The Acute Stress of the Confinement Process

At the time of moving into a farrowing crate (at 5 days before farrowing in this study), gilts showed an increase in blood cortisol [a biomarker used to quantify stress (65)] lasting at least 18 h (66). Gilts were not sampled after this time until they were close to farrowing, so it is uncertain how long it was after initial confinement before levels returned to baseline. It is unclear how much this response is affected by previous experience of confinement, as it has not been reported if there is an acute stress response in multiparous sows when moved into a farrowing crate. Furthermore, although EU legislation requires group housing in gestation, some animals may experience confinement in stalls during the first 4 weeks of pregnancy or short-term confinement in feeding stalls throughout gestation, which would reduce the novelty of being crated. It is also unclear what is the relative importance of the experience of confinement or of the stressors associated with being moved to a novel environment. Moving gilts and sows late (day 114 of gestation) into either free-farrowing pens or crates increased restlessness before farrowing in comparison with earlier entry into pens (days 95–105 of gestation) (67), but this study provided no data on animals entered early into crates. However, confining sows in crates at day 114 of gestation, or 2 days before farrowing, without moving them from their loose farrowing pens with straw did not increase salivary cortisol (28), and levels were actually higher in animals remaining loose-housed during the nest-building period.

Since elevated cortisol can antagonize the action of oxytocin (68), there is concern that placing sows in confinement at a time close to farrowing might adversely affect farrowing progress and increase the risk of stillbirths. In the study of Pedersen and Jensen (67), gilts introduced late to crates had longer interbirth intervals and a greater percentage of stillborn piglets than gilts introduced into pens, although there was no effect with older sows. Moving gilts into a crate once farrowing had commenced increased farrowing duration (68), as may closing gilts and sows into a crate at this point (69). In contrast, confining sows in crates at 2 days before farrowing without moving them from their loose farrowing pens did not increase farrowing duration (31).

#### The Impairment of Nest-Building Behavior

Confinement of sows that includes the 24-h period prior to the onset of farrowing will disrupt nest-building behavior, and this might also compromise farrowing performance (70). Thwarting the performance of normal nest building behavior by both confinement and lack of appropriate substrate resulted in higher levels of plasma cortisol in the active phase of the pre-farrowing period (66, 71, 72). However, environmental differences in cortisol level do not persist once farrowing is in progress and physiological aspects of parturition itself have an over-riding effect. The effect of frustrating nest building on cortisol is less marked in multiparous sows than in gilts, although still present (73), in accordance with other studies showing greater environmental sensitivity of farrowing behavior in primiparous animals (74). When the effects of confinement and nesting substrate are dissociated, it appears that it is the confinement that exerts the greatest effect on measures of HPA activation, possibly by preventing the important behaviors of walking and orientation during the nesting phase (75).

In a large study involving five different TC pen designs, Heidinger et al. (32) showed that the duration of nest building and the time active during the nest building phase were reduced in sows confined 1 day before expected farrowing in comparison with sows confined after completion of farrowing or never confined. Despite the reduced time spent active, confined sows showed an increased frequency of posture changes during the nest building phase. Hansen et al. (31) demonstrated a similar reduction in nest-building duration in the 24 h before farrowing when sows were confined in TC at 2 days before expected farrowing. Nest building behavior was performed with 32% greater frequency but 36% shorter bout length, suggesting that nest-building behavior was more fragmented as reported in earlier studies with confinement and lack of substrate (73). However, the confined sows showed only a non-significant increase in frequency of pen-directed oral behaviors, which might be indicative of frustration, and no associated difference in posture changing frequency over this period (31). In both studies, nest building substrate was provided in the form of straw. In a study where no nesting substrate was provided, confining sows from 6 days prior to expected farrowing resulted in increased nosing of crate features, although not bar biting, during farrowing in comparison with sows confined after completion of farrowing (47).

The consequences of the time of onset of TC for the farrowing process have also been inconsistent. Yun et al. (69) showed an increase in farrowing duration when confinement was imposed after the birth of the first piglet in comparison with 7 days before expected farrowing, although they reported no difference in oxytocin levels associated with this intervention (76). Studies in which confinement was imposed shortly (1 day or less) before expected farrowing have reported no difference when compared to unconfined sows (11, 32). While some studies have recorded an increased farrowing duration in sows confined from more than 2 days prior to expected farrowing in comparison with sows that were never confined or confined only after completion of farrowing (23, 24), other studies have shown no difference (27, 31, 47), although the former two studies involved hyperprolific sows with much longer farrowings in both confined and unconfined conditions. In general, the consequences for the number of stillborn piglets have mirrored the effects on farrowing duration in the different studies, with three studies showing increased stillbirths in sows crated for more than 2 days before expected farrowing (23, 24, 47), although in the latter case, this was not associated with a longer farrowing duration or increased signs of birth stress (meconium staining, vigor) in newborn piglets. In contrast, six papers reported no difference in stillbirth rate (27–29, 31, 42, 46), with all except the last of these involving hyperprolific sows. In the case of the two studies in which confinement was imposed shortly (1 day or less) before expected farrowing, there was no differences in stillbirth rate when compared to unconfined sows (11, 42), although the former study reported significantly more sows requiring farrowing assistance when TC was imposed.

#### The Consequences for Piglet Survival

Altering the ability to express nesting behavior may also have implications for subsequent maternal behavior and piglet survival [as reviewed by Yun and Valros (70)]. Successful performance of nest-building behavior has been associated in some, although not all, studies with more careful behavior of sows toward their offspring, improved nursing behavior, and greater IgG concentrations in neonatal piglet serum. When the crate remained open until the completion of farrowing, Nowland et al. (47) found that both piglet weight gain and piglet blood glucose on the first day were increased in comparison with confinement from farrowing house entry, although colostrum yield and Ig content did not differ. Hales et al. (28) also reported that confinement prior to farrowing resulted in a decreased frequency of nursing bouts in the following 2 days. However, Hales et al. (29) reported that closing the crate on completion of farrowing resulted in a higher proportion of dead piglets having an empty stomach when compared to both no crating or confinement from 3 days prior to farrowing, suggesting that disturbance at this time might also adversely affect suckling in hyperprolific litters.

The possible benefits of improved maternal behavior in unconfined sows may be countered by the increased risk of crushing with unrestricted movement of sows in the neonatal period. Some studies involving smaller litter sizes (total born <12 piglets) have reported only a non-significant increase in mortality prior to piglet processing or on the first day of life when comparing sows crated from >3 days before farrowing with those where the crate remained open until completion of farrowing (47) or until just before farrowing (23). However, two other studies with moderate litter size (total born: 12–15 piglets) have shown a substantial reduction from the mortality seen in unconfined conditions when confining the sow shortly (<1 day) before expected farrowing (11, 32). Studies involving hyperprolific sows (total born > 16 piglets) have also shown very significant increases (averaging > 100%) in mortality prior to piglet processing when comparing non-confinement with early confinement (24, 27–29, 46). The comparison of confinement shortly after completion of farrowing with confinement prior to farrowing is less conclusive, with two studies showing comparable low mortality (27, 28), while two others show little improvement over complete absence of confinement (29, 46). However, in all cases, the subsequent mortality in the period after litter equalization (to D3–D4 while the TC remained closed) showed no significant difference between early and post-farrowing crating.

Where the causes of mortality have been studied in more detail, it is deaths attributed to crushing that show the greatest increase in sows remaining unconfined (11, 24, 32). Crated sows show a reduced frequency of posture changes during farrowing (32, 47), although data are conflicting regarding the subsequent days. Studies report both fewer (28) or similar (32) frequency of posture changes when comparing sows confined before farrowing with unconfined sows, and in some cases (32), but not others (47), an increased frequency when sows are confined shortly after completion of farrowing.

### The Timing of Release From Confinement

The choice of time of release from confinement involves weighing the benefits of increased physical and behavioral freedom for the sow, with associated beneficial consequences for the piglets, and the risks of increased mortality from crushing when the piglets are housed with an unconfined sow. There are also other practical management considerations, which include the ease of maintaining hygienic conditions within pens of limited size and the safety of staff when carrying out routine piglet tasks, which are not discussed in this review.

#### Benefits for the Sows

During the first 2 days after giving birth, sows in semi-natural conditions generally remain inactive in the nest (77). A similar lack of activity is also shown by sows in farrowing crates and loose pens during this period (27, 28). It might therefore be assumed that confinement in a crate during this period imposes little real frustration of motivation for movement. However, from D3 postpartum onwards, sows begin to leave the nest area for increasing periods of time and roam over increasing distances with the piglets starting to follow her from D4. On average after 6–9 days, the nest is abandoned, and the sow and litter rejoin the family group (77, 78). If the only reason for leaving the nest is to find food, since much of the sow's time away is spent foraging, the provision of food within the farrowing crate may negate this motivation to move. However, other reasons for leaving the nest site are likely to have evolutionary importance, including the maintenance of hygiene, the motivation for environmental exploration, and the need for reintegration with the wider social group. Furthermore, confinement in the crate prevents the normal social interactions between the sow and her piglets and limits her ability to avoid the vigorous suckling attempts of the piglets as they increase in size.

##### Sow Activity and Motivated Behaviors

Removal of confinement and the associated larger space should increase the general activity and may allow the expression of motivated behavior such as exploratory behavior. Six research papers have reported on the effect of crate opening [on D3 (26); D4 (20); on D4 or D7 (17); on D7 (38); on D7 or D14 (40); on D10 (79)], on sow activity (i.e., number of postural changes, proportion of standing, walking, and lying) compared to permanent crating (Table 3). Findings from three papers showed an increase in general activity only during the first days post-crate opening compared to permanently crated sows (17, 26, 79). In contrast, two papers comparing similar housing conditions did not find any differences. It might be that the large observation interval or short overall observation times used in those studies were inadequate to detect differences [(20, 40), respectively]. Furthermore, there was no housing effect on the sow activity in subsequent lactation [D25, (26): weeks 3 and 4 of lactation (40)].

**Table 3:** Effect of crate opening on sow welfare.

	**Day of crate opening**	**Housing systems PC, permanent crating; TC, temporary crating; FF, Free Farrowing**	**Improvement (+), no change (=) or deterioration (–) associated with the use of TC vs. PC or FF**	**Results**	**References**
Activity	D3	TC vs. PC	+	Higher activity 24 h after crate opening on D4, but not on 25 in TC	(26)
	D4	TC vs. PC	=	No difference during the first 3 days after crate opening	(20)
	D4 or D7	TC vs. PC	+	Higher activity in TC over the first few days after crate opening	(17)
	D7	TC vs. PC	+	Higher activity in TC at D2 after crate opening	(38)
	D7 or D14	TC vs. PC	=	No difference from day of opening until weaning (D26).	(40)
	D10	TC vs. PC	+	Higher activity in TC from crate opening to weaning (D28)	(79)
Lying behavior	D3	TC vs. PC	=	No difference before vs. after crate opening	(35)
	D10	TC vs. PC vs. FF	+	Sows in FF or TC show more careful lying down behavior than sows in PC	(33)
Motivated behaviors	D4	TC vs. PC	+	More motivated behaviors in TC during the first 3 days after crate opening	(20)
	D4 or D7	TC vs. PC	+	More motivated behaviors in TC sows over the first 4 days after crate opening	(17)
	D5	TC vs. PC	+	More motivated behavior in TC sows over the first 7 days after crate opening	(41)
Stereotypies	D4	TC vs. PC	=	No differences from crate opening to D6	(20)
	D4 or D7	TC vs. PC	=	No differences over the first few days after crate opening	(17)
	D5	TC vs. PC	=	No differences from crate opening to D12	(41)
Nursing behavior	Completion of farrowing	TC vs. PC	+	Longer milk let-down in TC sows	(49)
			=	No difference in the number of nutritive and non nutritive nursings over the 3^rd^ and 4^th^ weeks of lactation	
	D3	TC vs. PC	=	No difference in the number of nursings, the number of nutritive, and non nutritive nursing on the day on the day of crate opening and D25	(34)
	D3	TC vs. PC	=	No difference in the nursing duration on D4, D11, and D18	(51)
	D4	TC vs. PC	=	No difference in the nursing duration over the first 3 days after crate opening	(20)
	D4 or D7	TC vs. PC	+	Longer nursing duration in TC after crate opening on D4 but not D7	(17)
	D5	TC vs. PC	+	TC sows spent a greater proportion of their time nursing during the first week post-partum	(41)
Interaction with piglets	D3	TC vs. PC	+	More interactions on D11 and D18 in TC	(51)
	D4	TC vs. PC	+	More interactions from crate opening to D6	(20)
	D4 or D7	TC vs. PC	+	More interactions over the first few days after crate opening in TC	(17)
	D5	TC vs. PC	+	More interactions from crate opening to D12 in TC	(41)
	D7	Before and after opening of TC	=	No differences in sniffing piglets	(38)
Skin lesions	D2	TC vs. PC vs. FF	+	TC and FF sows had fewer severe injuries on the udder and on the limbs than PC sows	(53)
	D3	TC vs. PC	–	More skin lesions on D4, D11, and D18 in TC	(51)
	D3, D4, or D6	TC vs. FF	–	More back and teat lesions when crated up to D6 vs. FF or crated up to D3	(44)
	D4 or 7	TC vs. PC	=	No differences after crate opening up to weaning (range: D28–D35) for udder and body lesions	(17)
			+	Fewer teat lesions on D21 in TC	
	D4 or D7	TC vs. PC	+	Lower risk for teat lesions in TC sows at D19	(18)
	D7 or D14	TC vs. PC	=	No differences on D7, D14 and D25	(40)
Stress response	During or after completion of farrowing	TC vs. PC	=	No differences in plasma cortisol 24 h prior to farrowing until the last born piglet	(47)
	D3	TC vs. PC	=	No difference on the day of opening and on D25	(26)
	~D5 or ~D18	TC vs. PC	+	Positive correlation with duration of confinement (end of lactation, D23) in TC- parities 3–8	(45)
	D4 or D7	TC vs. PC	Not conclusive	Not conclusive	(17)

*D refers to the number of days from farrowing day, which may be D0 or D1 (not always indicated in the papers)*.

King et al. (38) argued that posture changes may be less controlled after crate opening and therefore faster, increasing the risk of crushing as piglets have less time to escape. This was not supported by two studies focusing on sow-lying behavior that found that the duration of lying down and the proportion of piglets near the sow (in the danger zone) before lying down did not differ 24 h before and 24 h after crate opening (35) and that older piglets are stronger and can escape when the sow is lying down (80). Höbel et al. (33) observed that sows in zero-confinement housing or loose from D10 showed a more careful lying down behavior than sows in the crate. However, it is not clear to what extent the lying down behavior of the sow is influenced by pen design features (rails or sloped walls), which should allow more careful lying down (35) or whether sows were lying down in the middle of the pen without any support, which might be more dangerous (81).

Three papers focused on the question of whether sows provided with more space showed more exploratory behavior (e.g., pawing and rooting) after removal of confinement on D3 (41), D4 (20), and on D4 and D7 (17) (Table 3). All studies reported a higher proportion of exploration by sows after crate opening when compared to farrowing crates. Surprisingly, even though Chidgey et al. (20) did not find any effect of housing on the proportion of the general activity (e.g., time standing), they reported a higher proportion of rooting the floor after crate removal. This result suggests that the duration of the general activity is not a good proxy for all motivated behaviors such as exploration of the environment. It is important to highlight a limitation in all papers: the studies collected behavioral data over only a few days post-crate opening [3 days in Chidgey et al. (20); 7 days in Loftus et al. (41); and 4 days in Ceballos et al. (17)] and over brief periods per day. Thus, it is not possible to conclude from the present findings whether the increase in exploration after crate opening is only due to novelty or whether the provision of more space alone has a long-term effect (e.g., up to weaning) on satisfying the sow motivation to explore.

##### Sow–Piglet Interactions

In contrast to farrowing crates, TC pens allow more sow–piglet interactions based on the provision of more space and free movement once opened. Allowing sows and piglets to freely make nose contact may improve mother–young relationships and piglets' development, possibly benefiting animal welfare and productivity (82, 83). Five papers observed sow–piglet interactions after crate opening on D3 (51), on D4 (20), on D5 (41), on D4 or D7 (17), and D7 (38) and compared these with permanent crates (Table 3). Most of the available studies (four out of five) reported an increase in sow–piglet interactions after removal of confinement (17, 20, 41, 51), whereas one paper did not find any housing effect (38). A novelty effect was suggested by Ceballos et al. (17), where an increase in these interactions was reported only for the day of crate opening on D4 but not for the crate opening on D7 and nor until the end of the observation period on D8. In contrast, Singh et al. (51) reported a short- (D4) and long-term increase (D11 and D14) in social interactions between the sow and piglets compared to farrowing crates. However, the interactions were observed when the sows were lying during nursing, which suggests that the active part of the social interactions was initiated by the piglets.

#### Benefits for the Piglets

##### Piglet Behavior/Activity

Since the space available to the piglets is not changed when the sow is released from confinement, it might be anticipated that their behaviors would be less affected than those of the sow, and this has been shown to be the case for their exploratory behavior (36). However, the changed configuration of the space may give different behavioral opportunities, and the possibility for less-restricted interaction with the sow may beneficially change their social environment, since time spent playing is increased in the period following TC opening on D3 or D5 when compared to crates (41, 51). The level of activity may also be increased in later lactation (D18 and 25) in TC conditions compared to crates (33), but this has not always been observed (26). Chidgey et al. (21) reported that piglets reared in pens with TC spent more time inactive at the sow's udder when she was lying, more time inactive in the creep area and less time active in open areas when the sow was standing compared to piglets reared in crates when observed over the first 2 days post-opening (D5–D6, no further days were observed). The authors suggested that the sow is considered by the piglets as a source of comfort, warmth, and nutrition, but once allowed freedom of movement, she may not be as available as when housed in crates. Thus, piglets move to the creep for thermal comfort when she is standing and keep close to her when she is lying. However, an earlier study by the authors (20) found that, over the same period of time, piglets in pens with TC spent less time inactive in open areas than piglets in crates, while the amount of time piglets spent in the creep area did not differ between the two systems. Both pen design and thermal conditions are likely to affect piglet location preferences in individual studies. Pig-directed behaviors, including time spent massaging the sow and agonistic behavior toward other piglets, were both reduced when comparing TC until D5 with permanent crating (41), while manipulation of other piglets was also reduced with TC until D3 (51). In contrast, Kinane et al. (36) found no difference between TC until D4 and permanent crating in interactions with the sow, interactions with other piglets or agonistic behavior, although, interestingly, there was a tendency for less tail and ear biting following crate opening. Mack et al. (43) reported that piglets in permanent crating or TC spent less time touching (non-aggressive interactions) their companion piglets than in zero confinement. Controlled tests of piglet fearfulness/exploration in open field, human interaction, and startle tests have generally shown no difference between piglets from TC, permanent crating, and zero-confinement systems (36, 43). It should be noted that the TC systems in all these studies were reasonably basic, and no enrichment (e.g., straw) was provided. A larger and more diverse space (e.g., different flooring and functional areas) and provision of enrichment material has been shown to increase piglet activity, particularly locomotor and social play (84).

##### Piglet Performance

A widely reported benefit of non-crate farrowing and lactation systems has been a better growth rate and weaning weight of piglets (85). It has been suggested that this might result from prolonged benefits of zero confinement during nest building and a subsequent influence on colostrogenesis and then milking ability (70). It could also reflect the greater space in non-confinement situations, which allows better sow–piglet interaction and less restricted access to the udder during nursing.

*Nursing and suckling behavior*. The quality of nursing behavior is key to milk transfer. A higher number of nursings with milk ejection increases the milk intake, and a higher proportion of nursing without milk ejection decreases it (86, 87). Removing the physical restriction imposed by the crate may transiently increase the sow activity (26). Illmann et al. (34), thus suggesting that this might decrease the motivation to nurse and/or to release milk, leading in the short term to fewer nursings with milk ejection and more nursings without milk ejection. However, there might be a long-term positive effect on the course of nursings before weaning. It has been shown that nursing and suckling behaviors were calmer at weeks 2 and 4 when sows were loosely housed compared to permanent crates, and that sows terminated fewer nursing bouts and allowed the piglets to perform longer post-suckling udder massage (49). Three papers reported no differences in nursing behavior after crate opening on D4 [(20), observations on D4–6; (51), observations on D4, D11, and D18; (34), observations on D4 and D25]. Notably, Illmann et al. (34), in line with Pedersen et al. (49), where crate opening occurred after completion of farrowing, reported that the number of nursings with and without milk ejection did not differ between sows in farrowing crates or TC over the first 24 h after crate opening or over the last 2 weeks of lactation, indicating that milk transfer was not impaired (86). In contrast, three studies reported an effect of housing conditions on nursing behavior. Loftus et al. (41) observed that sows spent a greater proportion of their time nursing their piglets during the first week after crate opening on D5 compared to those in farrowing crates. Compared to farrowing crates, Ceballos et al. (17) reported a longer nursing duration immediately after crate removal on D4, but not on the following days (D5–D8), while Pedersen et al. (49) observed longer milk let-down during the last week of lactation in sows loose from the completion of farrowing. Altogether, the same duration of nursing or even a longer duration indicates that the nursing motivation is not impaired after crate opening compared to farrowing crates. When considered from the perspective of piglet suckling behavior, opening the crate may increase the space available around the udder, thus giving better access to the teats. This may result in less fighting for teats, fewer associated lesions in face and joints, and may also contribute to a more stable teat order. Kinane et al. (36) noted that piglets in a TC system were observed more often at the udder than those in a farrowing crate system. Furthermore, Chidgey et al. (21) reported that piglets in pens with TC massaged the udder more when the sow was lying when loose (D4–D6) compared to when crated (D1–D3), while no difference between these periods was observed in piglets of permanently crated sows. The authors suggested that the piglets may have compensated for a reduced opportunity to access the udder given that sows were more active once loose. In contrast, no difference in piglets missing milk ejection was observed when the crate was opened on D3 compared to permanent farrowing crating (34, 51). However, later in lactation, Singh et al. (51) reported that more piglets of sows confined until D3 were displaced during nursing in lactation pens on D11 and missed nursing bouts on D11 and D18 compared to piglets of sows permanently confined. Greater piglet competition may have been the reason, but it was not measured directly by the authors. An increase in skin lesions may be used to indicate more fighting among litter mates, especially during suckling when litter competition occurs. No differences in skin lesions were reported by Singh et al. (51) on the day after crate opening or during the second and third weeks of lactation in piglets of sows confined until D3 compared to those of permanently crated sows. Furthermore, the findings of Illmann et al. (34) do not support the hypothesis that crate opening leads to greater piglet competition during suckling. The authors found no difference in the proportion of piglets fighting during pre- and post-suckling udder massages and the proportion of piglets missing milk ejection between permanent and TC environment.

*Piglet growth*. Evidence for a benefit of opening the crate to piglet growth rate is inconsistent. While one study has reported poorer growth rate when comparing TC to permanent crating [D4: (19)], most have found no difference (13 different papers with crate opening between D2 and D18; Table 4). Five studies have reported a positive effect of crate opening on weight gain of piglets at weaning. Pedersen et al. (49) and Chidgey et al. (22) found that piglets weaned from pens where the sows were confined, until completion of parturition or until D4, respectively, were heavier (+0.8 and 0.2 kg) than those weaned from farrowing crates. Farmer et al. (25) reported an increased body weight (+0.16 kg) in piglets from sows loose from D3 compared to those housed in farrowing crate, but only during the third week of lactation. Nowland et al. (47) found that piglets born from sows confined after farrowing until D10 had higher weaning weight (+0.2 kg) than piglets born to sows confined from before farrowing to D10. Oostindjer et al. (48) found that piglets of sows crated from D0 to D4 tended to have better growth between D15 and weaning compared to those of sows in farrowing crates from D0 to weaning. It has been suggested that better nursing behavior [nursing duration and/or frequency (49, 88)] or a greater stimulation of solid food intake (48) could explain the increase in piglet body weight in loose environments. These hypotheses remain speculative, as none of the studies measured sow nursing behaviors nor measured piglet feed intake. Interestingly, an impact beyond the weaning phase has been observed. Kinane et al. (36) found only a tendency for heavier weights at D14 and D21 in lactation of pigs raised in a loose pen from D4 compared to those raised in farrowing crate, but by slaughter weight, these differences were significant with TC raised pigs being 4 kg heavier than crate raised pigs.

**Table 4:** Effect of crate opening on piglet welfare.

	**Day of crate opening**	**Housing systems PC, permanent crating; TC, temporary crating; FF, free farrowing**	**Improvement (+), no change (=) or deterioration (–) associated with the use of TC vs. PC or FF**	**Results**	**References**
Mortality	Completion of farrowing	TC vs. PC	=	No difference at weaning (range: D25–D28)	(49)
	D2	TC vs. PC vs. FF	–	Piglet mortality at weaning (D28) greater in TC and FF compared to PC when housed on slats	(50)
			=	No difference at weaning (D28) when housed on straw beeding	
	D3	TC vs. PC	=	No difference on the day of opening and on D25	(26)
	D3	TC vs. PC	+	Decrease in piglet mortality in TC when sows were crated and after crate opening, but only for sows of parity 3–4	(11)
	D3	TC vs. PC	=	No difference at weaning (range: D19–D26)	(51)
	D3 or D7	TC vs. PC	=	No difference at weaning (D26.4 ± 0.1)	(24)
	D4	TC vs. PC vs. FF	=	No difference from crate opening to D7	(27)
	D4	TC vs. PC vs. FF	+	Higher mortality in FF sows from crate opening to weaning (D28) compared to TC or PC sows	(29)
	D4	TC vs. PC	–	Increase in overall mortality in TC, but mortality lower before crate opening and higher after crate opening	(22)
	D4	TC vs. PC	=	No difference at weaning (D28)	(19)
	D4	TC vs. PC	=	No difference at weaning (D26.5 ± 1)	(36)
	D4	TC vs. PC	=	No difference at weaning (D26 ± 1)	(42)
	D4 or 7	TC vs. PC	–	Increased mortality in TC on D4 but not D7	(18)
	D4 or D7	TC vs. PC vs. FF	=	No difference from D1 to D10	(46)
	D5	TC vs. PC	=	No difference at weaning (D28)	(41)
	D5	TC vs. PC	=	No difference at weaning (D28)	(23)
	D5–17	TC vs. PC	=	No difference at weaning (D28)	(52)
	~D5 or ~D18	TC vs. PC	+	Positive relationship between the duration of confinement and piglet mortality	(45)
	D7 or D14	TC vs. PC	=	No difference at weaning (D26)	(40)
	D10	TC vs. PC	=	No difference at weaning (D28)	(33)
	D11	TC vs. PC	–	Higher mortality from day of opening until weaning (D28) in TC	(16)
	D14	TC vs. PC vs. FF	=	No difference after crate opening	(43)
			–	Lower mortality until crate opening in TC than in FF	
Suckling behavior	D3	TC vs. PC	=	No difference in piglets missing milk ejection on the day of crate opening	(34)
	D3	TC vs. PC	=	No difference in the number missing milk ejection on the day of crate openin	(51)
			–	More piglets displaced and missed milk ejection on D11 and D18 in TC	
	D4	TC vs. PC	+	Piglets in TC pens massaged the udder more when the sow was lying when loose compared to when crated	(21)
	D4	TC vs. PC	+	Increase time spent at the udder in TC from D4 to weaning (D26.5 ± 1)	(36)
Growth	Completion of farrowing	TC vs. PC	+	Higher body weight in TC piglets at weaning (range: D25–D28)	(49)
	D2	TC vs. PC vs. FF	=	No difference at weaning (D28)	(50)
	D3	TC vs. PC	+	Higher body weight in TC during the 3rd week of lactation	(25)
	D3	TC vs. PC	=	No difference on the day of crate opening and on D25	(26)
	D3	TC vs. PC	=	No difference on the day of crate opening and at weaning (range: D19–D26)	(51)
	D3 or D7	TC vs. PC	=	No difference on the day of crate opening and at weaning (D26)	(24)
	D4	TC vs. PC	+	Higher weight gain at weaning (D28) in TC	(22)
	D4	TC vs. PC	–	Lower weight gain piglets from gilts born and raised in pens compared to born and raised in crates or other combination of treatments.	(19)
	D4	TC vs. PC	+	Tendency for heavier weights in TC on D14 and D21, and heavier piglets at slaughter	(36)
	D4	TC vs. PC	=	No difference at weaning (D26 ± 1)	(42)
	D4 (D0–D4)	TC vs. PC	+	Better growth between D15 and weaning (D29.2 ± 2.7) in TC	(48)
	D5	TC vs. PC	=	No difference at weaning (D28)	(23)
	D5	TC vs. PC	=	No difference at weaning (D28)	(41)
	~D5 or ~D18	TC vs. PC	=	No difference at weaning (D23 ± 1)	(45)
	D5–D17	TC vs. PC	=	No difference at weaning (D28)	(52)
	D7 or D14	TC vs. PC	=	No difference on the day of crate opening and at weaning (D26)	(40)
	D10	TC vs. PC vs. FF	=	No difference at weaning (D28)	(33)
	D10	TC vs. PC	+	Higher weight gain at weaning (D21) in TC	(47)
	D11	TC vs. PC	=	No difference on the day of crate opening and at weaning (D28)	(16)
	D14	TC vs. PC vs. FF	=	No difference after crate opening	(43)
			+	Greater weight until crate opening in TC compared to FF (TC not different than PC)	
Activity	D3	TC vs. PC	=	No difference in activity on D5 or D25	(26)
	D3	TC vs. PC	+	Increased play behavior in TC; reduced manipulation of penmates	(51)
	D4	TC vs. PC	+	Decreased inactivity in open areas in TC	(20)
			=	No difference in time in creep area	
	D4	TC vs. PC	Uncertain	Increased time inactive at udder, in the creep and decreased activity in opens areas in TC on D5–D6	(21)
	D4	TC vs. PC	=	No difference in interaction with sow, interaction with other piglets and agonitic behvaiour or fearfulness/exploration in controlled tests.	(36)
	D5	TC vs. PC	+	Increased play behavior in TC; decreased pig directed behaviors in TC	(41)
	D10	TC vs. PC vs. FF	+	Increase activity on D18 and D25 in TC	(33)
	D14	TC vs. PC vs. FF	=	No difference in activity or fearfulness/exploration;	(43)
			+	Piglets in PC or TC spent less time touching (non-aggressive interactions) their companion piglets than in FF	
Skin lesions	D3	TC vs. PC	=	No differences the day after opening or during the 2nd and 3rd weeks of lactation	(51)
Stress response (cortisol levels)	~D5 or ~D18	TC vs. PC	+	Positive correlation with duration of confinement (end of lactation, D23 ± 1)	(45)

*D refers to the number of days from farrowing day, which may be D0 or D1 (not always indicated in the papers)*.

**Figure 2:**
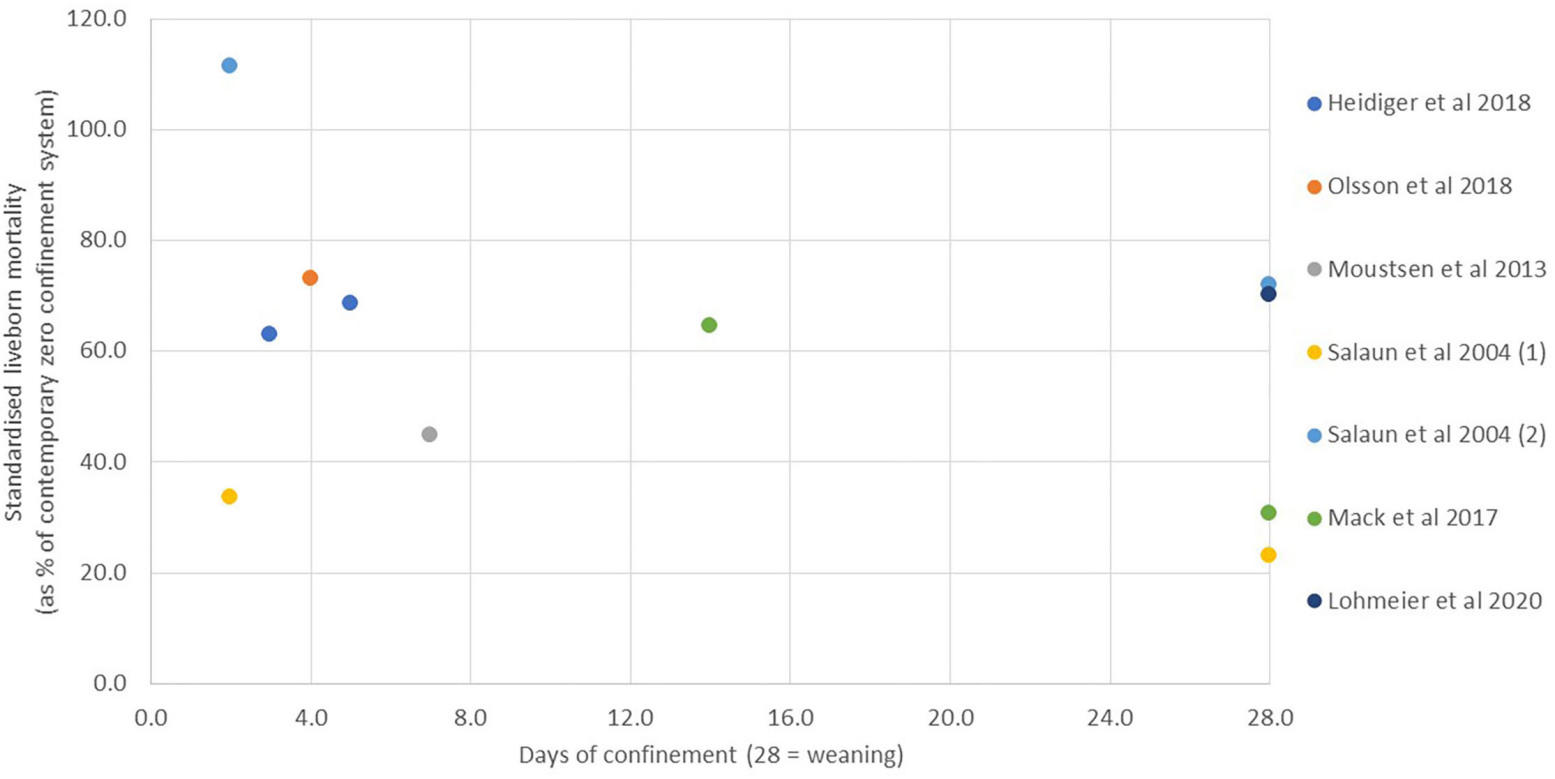
Liveborn mortality in temporary crating systems from birth to weaning compared to systems with zero confinement (values are given as the reported mean for temporary crating when expressed as a % of the contemporary zero-confinement treatment).

**Figure 3:**
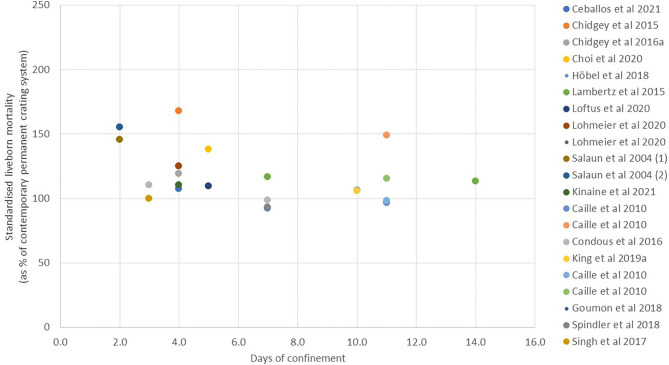
Liveborn mortality in temporary crating systems from birth to weaning compared to systems with permanent crating (values are given as the reported mean for temporary crating when expressed as a % of the contemporary permanent crating treatment).

*Piglet mortality*. The greatest concern about changing from a fully crated to a TC system is that piglet mortality will be increased as a result of allowing the sow greater freedom. In TC studies where the cause of mortality has been determined, it has been reported that significantly more piglets are crushed with earlier opening of the crate (18, 36, 42). However, since the majority of liveborn piglet mortality in all systems is reported to occur in the first few days after farrowing [e.g., 62% within the first 2 days and >80% within the first 7 days (89, 90)], there is debate about the period of time for which it is necessary to confine the sow. There is a growing body of literature on this question, and the results are explored in Figures 2, 3. Since the absolute level of mortality varies widely between studies, reflecting differences in genetics, quality of environment, and management, in each case, the mean level of mortality reported for the TC treatment has been standardized within study by expressing it as a percentage of the mortality reported for the comparator treatment (complete non-confinement or complete confinement, taken as 100). In Figure 2, the data from studies comparing different periods of TC with no confinement at any time are summarized to compare the mortality of liveborn piglets from birth to weaning (or in one study to D10) in the TC treatments when the fully non-confined treatment is taken as a reference value of 100. All studies, with one exception [TC until D2 in a straw-based system with moderate litter size of 13–14 born alive (50)], show a reduction in mortality when some period of crating is imposed. Where multiple points exist within the same study, they generally show reduced mortality with prolonged confinement relative to short confinement.

In Figure 3, the data from studies comparing different periods of TC with full confinement until weaning are summarized to compare the mortality of liveborn piglets from birth to weaning in the TC treatments when the fully confined treatment is taken as 100. While the majority of studies shows TC to give numerically higher mortality than full confinement, in only a few of the studies was this statistically significant due to high variation and relatively small sample sizes. A linear regression based on available standardized values gives a trend line indicating decreasing mortality as duration of confinement increases (slope of standardized mortality against day of −2.45 +/– 1.18, R-sq 0.177, *p* = 0.051). However, this appears mainly attributable to values in the first 4–5 days, after which no further benefit is apparent. One extreme outlier to this trend, which has been omitted from the figure, is a single extreme result for 14-day confinement, which reported a standardized mortality of 210% [(43); non-significant effect with 19–30 sows per treatment group]. The trend in Figure 3 is also in contrast to the within-farm study of Morgan et al. (45), who analyzed mortality data from a sample of 77 sows with confinement period ranging from 3 to 24 days. In their study, linear regression indicated that for every day of restraint, survival rate (the number of weaned pigs out of the number of live piglets at the age of 3 days) significantly decreased by 0.4%. When considering within-study comparisons, illustrated in the figures, over what would appear to be the most critical threshold time of 4–7 days, the most comprehensive overall study of this question (32) compared a crate opening time of 3 or 5 days with ~160 sows per treatment distributed across five different TC pen designs and concluded that no reduction in mortality resulted from the additional 2 days of confinement (11 vs. 12% of mortality for D3 and D5, respectively). Similarly, Moustsen et al. (46), with 55 sows per treatment, found no improvement in survival when comparing release on D4 or D7. However, Ceballos et al. (18) did find a significant difference in mortality when comparing TC until D4 or D7 with 161–185 sows per treatment, although with an atypically high overall mortality level (27.8 vs. 23.9% mortality).

## The Interpretation of Pig Welfare in Different Farrowing Systems

The objective of the review was to assess whether TC offers overall welfare benefits when compared with both the current system of permanent crating of the sow and also with a zero-confinement option favored by public opinion. It is apparent from the preceding review that there are differences in both behavior and production measures between the systems, although comparisons are often complicated by variable pen designs and operating procedures. The interpretation of these differences for the welfare of the animals is not always simple, but objective measures of differences in health, stress physiology, or abnormal behavior might provide evidence.

### Measures of Health

Providing the sow with greater space and exercise might be anticipated to improve health and reduce the risk of injuries from pen fittings. Seven studies have investigated the effect of crate opening on the prevalence of body lesions in sows (Table 3). The largest study on health-related parameters was done by Maschat et al. (44), who analyzed the effect of five different types of temporary crating pens and the time of confinement removal pp (zero confinement, on D3, D4, or D6) on 24 different injuries (e.g., lameness, shoulder lesion, teat lesions, neck injuries) on weeks 1, 3, and 4. Crate opening on D6 led to more back and teat lesions than zero confinement or crate opening on D3. They also showed that different temporary crating pen designs may have different effects on body lesions. Ceballos et al. (17, 18) did not find any effect of crate opening (on D4 or D7) on udder and body lesions or lameness until weaning, compared to farrowing crates. However, the authors reported less teat and udder lesions in the third week post-partum in sows housed in TC compared to sows remaining in closed crates. The results are in line with the findings of Lambertz et al. (40) where crates opened on D7 or D14 did not affect the number of body lesions on D7, D14, and D25. Conversely, Singh et al. (51) observed more body lesions on D4, D11, and D18 in sows confined until D3 compared to those of permanently crated sows. Similarly to Ceballos et al. (17, 18), Verhovsek et al. (53), and Lohmeier et al. (42) found a higher incidence of teat lesions on the third and fourth weeks of lactation in crated sows compared with sows loose from D2 or D4. Altogether, the results do not indicate a clear beneficial effect of crate opening on body lesions or lameness in sows after crate opening compared to sows housed in permanent crate or free farrowing.

From the perspective of the piglets, the most significant health parameter is absence of death or injury from crushing by the sow. Overall, most studies (12 out of 21) reported no statistically significant differences in piglet mortality rates between sows housed in temporary crates (opening D3–D14) and permanent farrowing crates (19, 23, 24, 26, 27, 40–42, 46, 49, 51, 52) or zero confinement (33) (Table 4). Four studies observed a decrease in piglet mortality in TC. Morgan et al. (45) found a negative relationship between the duration of confinement and piglet survival, reporting that for any additional day in restraint of the sows (3–23 days, i.e., permanent crating), piglets' weaning rate (number of piglets weaned relative to number alive at D3) decreased by 0.4%.

Olsson et al. (11) found a lower preweaning mortality in TC (until D3) compared to loose housing but only for sows of parity 3–4, suggesting an influence of sow maternal experience or physical conditions. Mack et al. (43) reported lower piglet mortality rates in TC compared to zero confinement during the crating period, but the difference was no longer seen from crate opening to weaning. When reported by time periods, piglet mortality was found to be similar (29) or lower (27) in TC compared to loose housing during the crating period but higher from crate opening to weaning (29) or similar from crate opening to D7 (27) after litter equalization.

Other studies have reported an increase in pre-weaning piglet mortality once the sows were no longer restricted by a crate compared to sows permanently crated [D4 (22); D4 or D7 (18) but not when loose from D7; D11 (16)]. King et al. (38) also recorded higher mortality rates post- vs. pre-crate opening. However, farrowing crates were not used as a control treatment. Even though higher prevalence of crushing events was often found to lead to higher piglet mortality [e.g., (11, 18, 22, 41)], it was not the case for all studies [e.g., (24, 52)]. In conclusion, the overall findings suggest that opening the crate after the vulnerable first few days does not impair pre-weaning piglet mortality compared to permanent crating and that TC reduces mortality when compared to free farrowing systems.

### Measures of Stress Physiology

When comparing TC to zero confinement, the consequence of thwarting nest building behavior for sow physiology has been ambiguous. In the study of Nowland et al. (47), no difference in plasma cortisol, measured hourly between 24 h prior to farrowing through until the last piglet was born, was seen between sows where the crate remained closed throughout or was open until completion of farrowing, although sows in crated conditions that had a prolonged farrowing showed elevated plasma cortisol once farrowing exceeded 5 h duration. Danish studies have shown higher salivary cortisol levels during the nest-building period in sows remaining unconfined in pens than in those where TC was imposed 2–3 days before expected farrowing (28, 31) and suggested that this might reflect greater activity in the unconfined sows, since exercise is known to activate the HPA axis (91). When considering the welfare of the sow during lactation, confinement in a farrowing crate may cause frustration and chronic stress, as many of the sow's motivated behaviors are constrained. Removal of confinement is thus expected to decrease cortisol levels. Ceballos et al. (17) measured salivary cortisol concentrations on the first day post-opening (D5) and found higher levels in sows loose from D4 compared to crated sows (to be loose on D7). However, no differences were found when compared to other groups of crated sows (permanently crated) on D5, which makes this result inconclusive. Focusing on a later point in lactation, Morgan et al. (45) reported lower cortisol concentrations before weaning at around D23 in sows subjected to a short period (up to D3–D10) of confinement compared to those confined for a longer period (up to D13–D23), but only in sows of parities 3–8. Investigating both short- and long-term effects of TC on cortisol concentrations, Goumon et al. (26) reported no influence of crate opening on D3 on salivary cortisol concentrations 24 h after opening of the crate nor in later lactation (D25). These findings reflect the unclear effect of the absence of confinement found in both early and late lactation. Some studies found no differences in salivary and plasma cortisol concentrations during the first week of lactation between sows housed in crates and permanent loose housing (92, 93), while others found either greater (2) or lower cortisol levels (28) in confined sows. Later in lactation, research suggests that confinement leads to chronic stress. Cronin et al. (92), Jarvis et al. (94), and Yin et al. (95) all found higher plasma cortisol levels in crated sows compared with sows housed in pens only at the end of lactation (D28, D29, and D35, respectively). This may reflect the parent–offspring conflict, whereby sows are motivated to wean their piglets, but have limited control over the contacts with their litter (96). Yet, recent research found no effect of permanent crating on hair cortisol compared to loose housing at mid and end of lactation (97). Stress caused by crating may be transferred from the sow to her piglets by a combination of impaired maternal behavior and higher cortisol concentration present in milk. Morgan et al. (45), who found a positive correlation between the duration of confinement (D3 to entire lactation) during lactation and hair cortisol concentrations in sows, reported a similar effect in piglets, suggesting increased stress levels in late lactation (D23) in piglets born to sows permanently crated.

Overall, research available shows inconclusive findings as to whether crate opening influences the cortisol response of sows and piglets in the short and long term.

### Measures of Abnormal Behavior

When highly motivated behaviors are prevented, animals may show the development of abnormal behaviors. Behaviors like repetitive bar biting and vacuum chewing are considered as stereotypies and are indicative of impaired welfare when observed for more than 10% of the time (98, 99). Opening the crate increases the space available and allows the sow to move freely within the pen. This may result in fewer stereotypies compared to farrowing crates (63, 70, 100, 101), as it could allow the sow more opportunities to express components of foraging behavior (e.g., locomotion). The three studies reporting on the occurrence of bar biting have shown no effect of housing on the proportion of time sows spent biting pen/crate fixtures in farrowing crates compared to TC pens after confinement removal on D4 (20), on D5 (41), and on D4 or D7 (17). Furthermore, sows performed bar biting in <10% of the observed periods. These findings suggest that fixture biting observed in sows is not indicative of compromised welfare in pen and crates. However, all studies observed sows only 3–4 days after crate removal. Consequently, it is not clear whether a housing effect might have occurred later in lactation. The same studies found no effect of housing on the proportion of vacuum chewing (17, 20, 41). However, a time effect on the proportion of vacuum chewing was detected in one study (20). The prevalence of vacuum chewing exceeded 10% from D4 in pens and from D2 in crates. This is most likely attributed to the absence of rooting material in both systems (20). This indicates that vacuum chewing may not be solved by larger space but rather by the provision of manipulable materials (95, 102). Altogether, the available studies suggest that provision of more space did not reduce stereotypies.

## Methodological Limitations and Knowledge Gaps

Review of the published studies on TC has highlighted several methodological considerations that should be considered in the design of future studies and a number of important areas where research is still lacking.

### Behavioral and Physiological Data Collection

Behavioral data have been collected in some studies over only brief periods per day [e.g., 100 ×30 s observations per day, (20, 21); or 15 min bi-hourly six times per day (41)], which may have reduced the reliability of observations. This contrasts with other studies in which behavior was collected at short interval over a longer period after crate opening [e.g., 5-min interval over 24 h (26); 2-min interval over 12 h (17); or 10-min interval for a period of 3 weeks (40)]. Findings on the cortisol response remain mainly inconclusive (Section Measures of Stress Physiology). The validity of using cortisol to measure stress levels associated with confinement may be questioned (65). Physiological increases in plasma or salivary concentrations of cortisol around farrowing may also mask variations due to environmental or psychological factors. Furthermore, the discrepancy between the studies for both sow and piglet cortisol response to TC may also reflect the variation in matrixes used and in the confinement duration, along with the low number of cortisol samples.

### Statistical Power of Comparisons

Many of the published studies are based on relatively small sample sizes. As a result, firm conclusions about differences in measures with a high coefficient of variation are often not possible (103). This is particularly relevant in the case of interpretation of piglet mortality. Based on our descriptive analysis, piglet mortality seems to decrease as duration of confinement increases, although this is attributable to the mortality due to crate opening during the first week. It is important to highlight that the cause and timing of death was not always identified in the cited studies. Providing this information could help better identify whether opening of the crate was the determining factor of piglet death. Most studies [e.g., (16, 19, 41, 45, 51)] provided mortality rates averaging piglet death from crate opening to weaning. Only few provided detailed mortality rates, for instance on the day of crate opening (26) or on a weekly basis (40). In addition, most studies used litter equalization within the first days post-farrowing. While this provides standardization when comparing TC to permanent crating, it may mask differences in mortality prior to equalization, especially when comparing TC to loose housing. Therefore, it is important to provide more details on piglet mortality e.g., stillborn, dead before litter equalization, and dead after litter equalization [e.g., (46)]. Finally, as pointed out by Damm et al. (104), a minimum of just over 150 farrowings per treatment are needed to detect a difference in piglet mortality of 0.2 piglet per litter, on the basis of the variation in piglet mortality found in previous studies. Therefore, with the exception of Hales et al. [(29); *N* = 658–682 sows/treatment] and Chidgey et al. [(22); *N* = 338–394 sows/treatment], the significance of the mentioned results on piglet mortality remains questionable. The number of farrowing used in the other studies ranged from 12–13 (26, 41) to ~130–185 per treatment (18, 32, 38, 39).

### Biological Relevance of Findings

When statistically significant treatment effects are found, it is important to look at the biological relevance of the findings to make informed interpretation. Regarding sow activity, Goumon et al. (26) found an increase of 10 and 20 min in time spent active and rolling, respectively, over the 24 h following crate opening on D4, between sows released from confinement and those remaining crated. Ceballos et al. (17) found a difference of ~10–15 min spent standing and a reduction of time spent inactive of ~7% translating to ~25 min, and a twofold increase in postural changes (~25–50 changes per day) over the 12 h following crate opening on D4 or D7, between sows release from confinement and crated sows. The observed small differences were to be expected, as time spent active, and especially exploring the environment and investigating the piglets, represent a small proportion of the daily time budget of sows [<10%, e.g., (17, 26, 40)]. Further research is needed to determine how significant such differences are for the welfare of the animals. It is also important to consider whether the absence of beneficial effects in some studies is due to the pen designs, which do not satisfy the needs of the sows (or piglets). Simply opening the crate may not be beneficial if the sows cannot then fulfill motivated behaviors (e.g., foraging, reintegration with herd), and lack of biologically relevant enrichment provision and space to perform important behaviors, such as play, will impact on piglet activity budgets (105). Research is needed focusing on the question of whether a more stimulating pen design can increase motivated exploratory behavior in the long term after confinement removal. Another research focus might be to allow longer foraging time and more social contacts with neighboring sows and piglets, in accordance with the natural behavior of the sow (77, 78).

### Crate Opening Time and Procedure

The definition of day of opening is another methodological challenge. While some studies simply mentioned that the crate was open on day X (24, 47, 51), others referred to the day of opening with the following expressions: confinement until day X (40) or confined to day X (44), implying that temporary crate was removed on day X. Others refer to confinement to day X having sows loose from day X + 1 (29). Most research papers refer to the day of crate opening having the day of farrowing as reference point. Yet, some studies defined the day of farrowing as day 0 (21, 26, 46), while many did not [e.g., (24, 41)]. In the latter case, it may then be assumed that the day of farrowing is considered as day 1. Hence, while crate opening occurred on D3 in Goumon et al. (26) or D4 in Moustsen et al. (46), the dates would be considered as D4 or D5, respectively, based on the definition crate opening used in other studies. In addition, it is not clear whether the reference point is the start or end of farrowing. This may be of importance given that farrowing environment may impact farrowing duration (2). Finally, while crate opening is mostly done on a specific day, it was sometimes defined as a range of days. Singh et al. (51) opened crates at an average of D3, ranging from D2 to D5. While Spindler et al. (52) opened between D5 and D17. Similarly, Maschat et al. (44) indicated that sows were crated at the end of farrowing to D4, resulting in a median duration of 2.92 days (min = 1.98, max = 3.65). In other studies, the range was not provided, e.g., King et al. (37) opened at approximately D10. In addition to a clear definition of the day of crate opening, the actual timing and crate opening procedure were often not well-defined in the papers presented in this review. It is a challenge to standardize opening techniques/procedures (e.g., day of opening or time of the day), as studies were done on different farms where farrowing management, workflow, and feeding schedules may have differed. Yet, this is of importance because procedure for opening may influence piglet mortality. King et al. (38) opened crates individually, either in the morning or afternoon at 7 days of age or all crates simultaneously once the average litter age reached 7 days. The authors showed that piglet mortality during the first 2 days after crate opening was reduced by opening crates for individual litters, rather than as a batch, more so in the afternoon, while only a statistical tendency for such a difference was observed over the full period from crate opening to weaning. The time at which the crate was opened was mentioned in few of the papers used in this review (17, 18, 26). Further research on the best procedure for crate opening to minimize associated risk of piglet mortality is required.

### Lack of Long-Term Data

In most studies, behavioral data collection has been limited to the day of crate opening or the first days after crate opening [e.g., (17, 20, 41, 46)]. Thus, long-term effects of removal of confinement remain unknown. While some studies have also collected data until mid- to late lactation, daily (40) or on specific days (26, 51), very few studies have looked at the effect of crate opening on sow and piglet behavior and performance beyond the suckling period (20, 36, 39). The importance of early-life experience in shaping future behavioral and physiological responses to challenges later in life is well-documented in mammals [for reviews—(106, 107)] including pigs (108). Studies specifically relating to the farrowing environment have mainly concentrated on systems where the sow is either crated or loose and/or the level of enrichment provided for the piglets [e.g., (109, 110)] rather than exploring TC systems *per se* and therefore, the added layer of the sow being partly confined, partly loose. Some papers have explored effects of TC vs. permanent crating on piglet post-weaning behavior (111), responsiveness to behavioral tests, and growth to finishing stage (36). There is also one transgenerational maternal behavior study following gilts born to mothers reared in either TC or permanent crating (19, 20). All reported positive effects of TC compared to permanent crating on the measures taken, but more studies are needed to be able to provide definitive conclusions about any long-term benefits.

Further studies in this area would be valuable given the importance placed on early-life experience for shaping long-term outcomes [including documented transgenerational effects influencing farrowing behavior—(19, 37)] and the potential for long-term benefits to offset the increased costs of transitioning from existing systems (112). Studies on the development of cognitive abilities in piglets are also needed, since confining the sows in farrowing crates may increase stress, therefore impairing piglets' cognitive abilities and performances (2, 113, 114).

## Conclusions

### Comparing Temporary Crating With Permanent Crating

The literature presented in this review paper suggests that sows may benefit in the short term from a reduction in confinement compared to permanent crating. Sows housed in TC increase their general activity, express more exploration behavior, and engage in more interactions with piglets during the day of opening. Yet, it remains unclear whether there are any longer-term beneficial effects (until or beyond weaning) due to the paucity of studies. Thus, this does not allow us to conclude whether the increase in motivation to engage in these behaviors is simply due to the novelty and larger surface of the available space or whether it would be sustained over time. Furthermore, while no detrimental effect was reported, it remains unclear whether the observed short-term benefits translate to other welfare indicators. Research findings indicate no reduction in the frequency of stereotypies or body lesions and do not provide clear answers regarding sow stress response when released from confinement. Yet, it is important to consider that TC allows greater freedom of movement for most of the time in the farrowing unit and, in some pen designs, the possibility to allocate different activities (rest, eat, eliminate) to different areas of the pens.

Research findings indicate that TC of the sow does not have detrimental effects on piglet performance compared to crates. Suckling behavior and growth performance of piglets after the sow is released from confinement remain mostly unaffected or, in some cases, improved. There are concerns that opening the crate may lead to an increase in piglet mortality as sow's dangerous posture changes are no longer reduced. Experimental findings indicate that failure to close the crate prior to farrowing or very early opening of the crate seems to significantly increase piglet mortality. However, when confinement is correctly timed during the most vulnerable period around farrowing, piglet mortality can be comparable to that with permanent crating. Finally, based on limited studies and limited pen designs, the effect of reduced confinement of the sows has limited beneficial effects on piglet social behavior, while it had no effect on their stress reactivity. The consequences for longer-term social and cognitive skills of piglets require further investigation.

### Comparing Temporary Crating With Zero Confinement

Confining the sow aims mostly at reducing postural changes that may lead to crushing of piglets and facilitating stockperson interventions to assist piglet survival. Thus, piglets may benefit from a short-term confinement of the sow, preventing possible increased risk of piglet mortality when sows are loose during farrowing and lactation. The limited available research indicates no effect of short maternal confinement on piglet growth performance, while the period of crating seems to contribute to reduce piglet mortality. More research is needed to investigate the possible hindrance that the farrowing crate may have on the behavioral interactions between the sow and the piglets during the crating period. Confinement during the period prior to farrowing may prevent nest building, an important and highly motivated behavior. Thus, confining the sow after farrowing may be the best option for sow welfare, despite the need for increased supervision by farmers. Yet, consequences of prevented nest-building behavior on sow physiology have been ambiguous. Furthermore, the impact of the time of onset of TC on the farrowing process and piglet mortality have been inconsistent, making a decision on when to confine the sow difficult.

### Future Research

There are methodological limitations and knowledge gaps that call for further research. The quality of the data from future studies is very important (e.g., adequate sample sizes and data collection periods, comprehensive reporting of methodological details, and performance outcomes) if they are going to effectively contribute to the evidence base upon which political and commercial decisions are being made.

## Author Contributions

The conceptual work was provided by SG led with support from GI, VAM, EMB, and SAE. Review work and manuscript drafting by SG, GI, VAM, EMB, and SAE. All authors contributed to the article and approved the submitted version.

## Funding

GI was supported by grant MZE-RO0718 from the Ministry of Agriculture of the Czech Republic. EMB was supported by the Scottish Government's Rural and Environment Science and Analytical Services Division (RESAS). VAM was partly funded by the Danish Pig Levy Foundation.

## Conflict of Interest

VAM worked for SEGES Danish Pig Research Center. The aim of the Danish Pig Research Center is to safeguard the interests of the Danish pig producers. The remaining authors declare that the research was conducted in the absence of any commercial or financial relationships that could be construed as a potential conflict of interest.

## Publisher's Note

All claims expressed in this article are solely those of the authors and do not necessarily represent those of their affiliated organizations, or those of the publisher, the editors and the reviewers. Any product that may be evaluated in this article, or claim that may be made by its manufacturer, is not guaranteed or endorsed by the publisher.

## Footnotes

^1^Verordnung des Bundesministers für Gesundheit, mit der die 1. Tierhaltungsverordnung geändert wird. Bundesgesetzblatt für die Republik Österreich BGBl. II Nr. 61/2012.

^2^ https://www.gesetze-im-internet.de/tierschnutztv/BJNR275800001.html

^3^Swedish Board of Agriculture's Regulations (SJVFS, 2019, p. 20).

^4^*Swiss Animal Protection Ordinance*. Available online at: https://www.blv.admin.ch/blv/en/home/tiere/tierschutz.html

## References

[1] Baxter EM Andersen IL Edwards SA. Sow welfare in the farrowing crate and alternatives. In: Špinka M, editor. Advances in Pig Welfare. Cambridge: Elsevier Ltd., Woodhead Publishing (2018). p. 27–72. 10.1016/B978-0-08-101012-9.00002-2

[2] Oliviero C Heinonen M Valros A Hlli O Peltoniemi OAT. Effect of the environment on the physiology of the sow during late pregnancy, farrowing and early lactation. Anim Reprod Sci. (2008) 105:365–77. 10.1016/j.anireprosci.2007.03.015 17449206

[3] Gu Z Gao Y Lin B Zhong Z Liu Z Wang C. Impacts of a freedom farrowing pen design on sow behaviours and performance. Prevent Vet Med. (2011) 102:296–303. 10.1016/j.prevetmed.2011.08.001 21880386

[4] Peltoniemi OAT Oliviero C. Housing, management and environment during farrowing and early lactation. In: Farmer C, editor. The Gestating and Lactating Sow. Wageningen Academic Publishers (2015). p. 1–452. 10.3920/978-90-8686-803-2_10

[5] Hales J Moustsen VA Nielsen MBF Hansen CF. Higher preweaning mortality in free farrowing pens compared with farrowing crates in three commercial pig farms. Animal. (2014) 8:113–20. 10.1017/S1751731113001869 24152336

[6] Pedersen LJ Malmkvist J Andersen HML. Housing of sows during farrowing: a review on pen design, welfare and productivity. In: Livestock Housing: Modern Management to Ensure Optimal Health and Welfare of Farm Animals. Wageningen Academic Publishers (2013). p. 93–111. 10.3920/978-90-8686-771-4_05

[7] Vandresen B Stadnick ECP Hötzel MJ. Freedom to move versus piglet crushing: citizens' attitudes towards farrowing housing systems. In: Proceedings of the Global Virtual Meeting of the International Society for Applied Ethology (2020).

[8] Weber R Burla JB Jossen M Wechsler B. Excellent performance with larger litters in free-farrowing pens. Swiss Agri Res. (2020) 11:53–8. 10.34776/afs11-53e

[9] Andersson E Frössling J Engblom L Algers B Gunnarsson S. Impact of litter size on sow stayability in Swedish commercial piglet producing herds. Acta Vet Scand. (2015) 58:3. 10.1186/s13028-016-0213-8 27207437 PMC4875734

[10] AHDB. 2019 Pig Cost of Production in Selected Countries. Kenilworth: Agriculture and Horticulture Development Board (2021). Available online at: https://ahdb.org.uk/knowledge-library/2019-pig-cost-of-production-in-selected-countries (accessed December 25, 2021).

[11] Olsson AC Botermans J Englund JE. Piglet mortality - a parallel comparison between loose-housed and temporarily confined farrowing sows in the same herd. Acta Agric Scand A Anim Sci. (2018) 68:52–62. 10.1080/09064702.2018.1561934

[12] Pig Research Centre. Action Plan Better Animal Welfare for Pigs. (2011). Available online at: https://en.fvm.dk/fileadmin/user_upload/FVM.dk/Dokumenter/Landbrug/Indsatser/Dyrevelfaerd_og_-transport/Svinehandlingsplan_engelsk_final.docx (accessed December 25, 2021).

[13] Fink Hansen C. Change experiences by a Danish policy influencer. In: Virtual Workshop “Freedom in Farrowing and Lactation“: Overcoming Barriers, Facilitating Change (2021).

[14] Baumgartner J. Country roundup. In: Virtual Workshop “Freedom in Farrowing and Lactation”: Overcoming Barriers, Facilitating Change (2021).

[15] Baxter EM Lawrence AB Edwards SA. Alternative farrowing systems: design criteria for farrowing systems based on the biological needs of sow and piglets. Animal. (2011) 5:580–600. 10.1017/S1751731110002272 22439955

[16] Caille ME Meunier-SAlaün MC Ramonet Y. Sows in loose farrowing system effects on performance and work conditions. Swine Days Res. (2010) 9–13.

[17] Ceballos MC Góis KCR Parsons TD. The opening of a hinged farrowing crate improves lactating sows' welfare. Appl Anim Behav Sci. (2020) 230:105068. 10.1016/j.applanim.2020.105068

[18] Ceballos MC Rocha Gois KC Parsons TD Pierdon M. Impact of duration of farrowing crate closure on physical indicators of sow welfare and piglet mortality. Animals. (2021) 11:969. 10.3390/ani11040969 33807217 PMC8065918

[19] Chidgey KL Morel PC Stafford KJ Barugh IW. The performance and behaviour of gilts and their piglets is influenced by whether they were born and reared in farrowing crates or farrowing pens. Livest Sci. (2016) 193:51–7. 10.1016/j.livsci.2016.09.011

[20] Chidgey KL Morel PC Stafford KJ Barugh IW. Observations of sows and piglets housed in farrowing pens with temporary crating or farrowing crates on a commercial farm. Appl Anim Behav Sci. (2016) 176:12–8. 10.1016/j.applanim.2016.01.00430616274

[21] Chidgey KL Morel PC Stafford KJ Barugh IW. Sow and piglet behavioral associations in farrowing pens with temporary crating and in farrowing crates. J Vet Behav. (2017) 10:91–101. 10.1016/j.jveb.2017.01.003

[22] Chidgey KL Morel PCH Stafford KJ Barugh IW. Sow and piglet productivity and sow reproductive performance in farrowing pens with temporary crating or farrowing crates on a commercial New Zealand pig farm. Livest Sci. (2015) 173:87–94. 10.1016/j.livsci.2015.01.003

[23] Choi Y Min Y Kim Y Jeong Y Kim D Kim J. Effects of loose farrowing facilities on reproductive performance in primiparous sows. J Anim Sic Technol. (2020) 62:218–26. 10.5187/jast.2020.62.2.218 32292929 PMC7142289

[24] Condous PC Plush KJ Tilbrook AJ van Wettere WHEJ. Reducing sow confinement during farrowing and in early lactation increases piglet mortality. J Anim Sci. (2016) 94:3022–9. 10.2527/jas.2015-0145 27482689

[25] Farmer C Devillers N Widowski T Massé D. Impacts of a modified farrowing pen design on sow and litter performances and air quality during two seasons. Livest Sci. (2006) 104:303–12. 10.1016/j.livsci.2006.04.010

[26] Goumon S Leszkowová I Šimečková M Illmann G. Sow stress levels and behavior and piglet performances in farrowing crates and farrowing pens with temporary crating. J Anim Sci. (2018) 96:4571–8. 10.1093/jas/sky324 30102369 PMC6247827

[27] Hales J Moustsen VA Devreese AM Nielsen MBF Hansen CF. Comparable farrowing progress in confined and loose housed hyper-prolific sows. Livest Sci. (2015) 171:64–72. 10.1016/j.livsci.2014.11.009

[28] Hales J Moustsen VA Nielsen MBF Hansen CF. The effect of temporary confinement of hyperprolific sows in sow welfare and piglet protection pens on sow behaviour and salivary cortisol concentrations. Appl Anim Behav Sci. (2016) 183:19–27. 10.1016/j.applanim.2016.07.008

[29] Hales J Moustsen VA Nielsen MB Hansen CF. Temporary confinement of loose-housed hyperprolific sows reduces piglet mortality. J Anim Sci. (2015) 93:4079–88. 10.2527/jas.2015-8973 26440187

[30] Hansen LU. Test of 10 Different Farrowing Pens for Loose-Housed Sows. SEGES Svine Produktion. Report No 1803. Copenhagen: SEGES Danish Pig Research Centre (2018).

[31] Hansen CF Hales J Weber PM Edwards SA Moustsen VA. Confinement of sows 24h before expected farrowing affects the performance of nest building behaviours but not progress of parturition. Appl Anim Behav Sci. (2017) 188:1–8. 10.1016/j.applanim.2017.01.003

[32] Heidinger B Stinglmayr J Maschat K Oberer M Kuchling S Baumgartner J. Summary of the Austrian Project “Pro-SAU”: Evaluation of Novel Farrowing Systems with Possibility for the Sow to Move. Copenhagen: SEGES Danish Pig Research Centre (2018).

[33] Höbel C Klein S Patzkéwitsch D Reese S Erhard M. A comparison of different farrowing systems. Part 2 Performance data and effects on the lying down behaviour of the sows and the activity of the piglets. Tierarztl Prax Ausg G Grosstiere Nutzt. (2018) 46:357–67. 10.15653/TPG-180484 30616274

[34] Illmann G Goumon S Šimečková M Leszkowová I. Effect of crate opening from day 3 postpartum to weaning on nursing and suckling behaviour in domestic pigs. Animal. (2019) 13:2018–24. 10.1017/S1751731118003750 30704543

[35] Illmann G Goumon S Chaloupkov á H. Assessment of lying down behaviour in temporarily crated lactating sows. Animal. (2021) 15:100130. 10.1016/j.animal.2020.100130 33573954

[36] Kinane O Butler F O'Driscoll K. Freedom to grow: improving sow welfare also benefits piglets. Animals. (2021) 11:1181. 10.3390/ani11041181 33924235 PMC8074778

[37] King RL Baxter EM Matheson SM Edwards SA. Sow free farrowing behaviour: experiential, seasonal and individual variation. Appl Anim Behav Sci. (2018) 208:14–21. 10.1016/j.applanim.2018.08.006

[38] King RL Baxter EM Matheson SM Edwards SA. Temporary crate opening procedure affects immediate post-opening piglet mortality and sow behaviour. Animal. (2019) 13:189–97. 10.1017/S1751731118000915 29733002

[39] King RL Baxter EM Matheson SM Edwards SA. Consistency is key: interactions of current and previous farrowing system on litter size and piglet mortality. Animal. (2019) 13:180–8. 10.1017/S1751731118000927 29720289

[40] Lambertz C Petig M Elkmann A Gauly M. Confinement of sows for different periods during lactation: effects on behaviour and lesions of sows and performance of piglets. Animal. (2015) 9:1373–8. 10.1017/S1751731115000889 25994275

[41] Loftus L Bell G Padmore E Atkinson S Henworth A Hoyle M. The effect of two different farrowing systems on sow behaviour, piglet behaviour mortality and growth. Appl Anim Behav Sci. (2020) 232:105102. 10.1016/j.applanim.2020.105102

[42] Lohmeier RY Gimberg-Henrici CGE Burfeind O Krieter J. Suckling behaviour and health parameters of sows and piglets in free-farrowing pens. Appl Anim Behav Sci. (2019) 211:25–32. 10.1016/j.applanim.2018.12.006

[43] Mack LA Rossini SP Leventhal SJ Parsons TD. Case study: differences in social behaviors and mortality among piglets housed in alternative lactational systems. Prof Anim Sci. (2017) 33:261–75. 10.15232/pas.2016-01564

[44] Maschat K Dolezal M Leeb C Heidinger B Winckler C Oczak M. Duration of confinement and pen-type affect health related measures of welfare in lactating sows. Anim Welf. (2020) 29:339–52. 10.7120/09627286.29.3.339

[45] Morgan L Meyer J Novak S Younis A Ahmad WA Raz T. Shortening sow restraint period during lactation improves production and decrease hair cortisol concentrations in sows and their piglets. Animal. (2021) 15:100082. 10.1016/j.animal.2020.100082 33509702

[46] Moustsen VA Hales J Lahrmann HP Weber PM Hansen CF. Confinement of lactating sows in crates for 4 days after farrowing reduces piglet mortality. Animal. (2013) 7:648–54. 10.1017/S1751731112002170 23190747

[47] Nowland TL Ernest WH van Wettere J Plush KJ. Allowing sows to farrow unconfined has positive implications for sow and piglet welfare. Appl Anim Behav Sci. (2019) 221:104872. 10.1016/j.applanim.2019.104872

[48] Oostindjer M Bolhuis JE Mendl M Held S Gerrits W van den Brand.. Effects of environmental enrichment and loose housing of lactating sows on piglet performance before and after weaning. J Anim Sci. (2010) 88:3554–62. 10.2527/jas.2010-2940 20622185

[49] Pedersen ML Moustsen VA Nielsen MBF Kristensen AR. Improved udder access prolongs duration of milk letdown and increases piglet weight gain. Livest Sci. (2011) 140:253–61. 10.1016/j.livsci.2011.04.001

[50] Salaün C Le Roux N Vieuille C Meunier-Salaün MC Ramonet Y. Effect of housing system on lactating sows and piglets behaviour and on their performances before weaning. Swine days Res. (2004) 36:371–8.

[51] Singh C Verdon M Cronin GM Hemsworth PH. The behaviour and welfare of sows and piglets in farrowing crates or lactation pens. Animal. (2017) 11:1210–21. 10.1017/S1751731116002573 27917741

[52] Spindler E Klein S Erhard M Reese S Patzkewitsch D. Field trial of an open pen – comparison of two different types of farrowing pens. Vet Pract Ausg G Large Anim Farm Anim. (2018) 46:283–90. 10.15653/TPG-180010 30340237

[53] Verhovsek D Troxler J Baumgartner J. Peripartal behaviour and teat lesions of sows in farrowing crates and in a loose-housing system. Anim Welf. (2007) 16:273–6.

[54] Glencorse D Plush K Hazel S D'Souza D Hebart M. Impact of non-confinement accommodation on farrowing performance: a systematic review and meta-analysis of farrowing crates versus pens. Animals. (2019) 9:957. 10.3390/ani9110957 31726676 PMC6912515

[55] Moustsen VA Lahrmann HP d'Eath RB. Relationship between size and age of modern hyper-prolific crossbred sows. Livest Sci. (2011) 141:272–5. 10.1016/j.livsci.2011.06.008

[56] Nielsen SE Kristensen AR Moustsen VA. Litter size of Danish crossbred sows increased without changes in sow body dimensions over a thirteen-year period. Livest Sci. (2018) 209:73–6. 10.1016/j.livsci.2018.01.015

[57] Moustsen VA Duus LK. Søers ”Rejse og Lægge sig” Bevægelse i Forskellige Farestier. Dansk Svine Produktion. Report No 733. Copenhagen: SEGES Danish Pig Research Centre (2006).

[58] Damm BI Vestergaard KS Schroder-Petersen DL Ladewig J. The effects of branches on prepartum nest building in gilts with access to straw. Appl Anim Behav Sci. (2000) 69:113–24. 10.1016/S0168-1591(00)00122-2 10906396

[59] Nannoni E„Aarnink AJA Vermeer HH Reimert I Fels M Bracke MBM. Soiling of pigs pens: a review of elimintive behaviour. Animals. (2020) 10:2025. 10.3390/ani10112025 33153115 PMC7693532

[60] Moustsen VA Poulsen HL. Pattegrises Dimensioner. Landsudvalget for Svin, Danske Slagterier. Report No 0432. Michael Conn P, editor. (2004).

[61] Moustsen VA Nielsen MBF. Dimensioner på 202 Danske Pattegrise Målt I En Besætning. SEGES Svine produktion. Report No 1727. Copenhagen: SEGES Danish Pig Research Centre (2017).

[62] Verdon M Morrison RS Rault JL. The welfare and productivity of sows and piglets in group lactation from 7, 10, or 14 d postpartum. J Anim Sci. (2020) 98:1–11. 10.1093/jas/skaa037 32005996 PMC7057952

[63] Andersen IL Vasdal G Pedersen LJ. Nest building and posture changes and activity budgets of gilts housed in pens and crates. Appl Anim Behav Sci. (2014) 159:29–33. 10.1016/j.applanim.2014.07.002

[64] Malmkvist J Pedersen LJ Kammersgaard TS Jorgensen E. Influence of thermal environment on sows around farrowing and during the lactation period. J Ani Sci. (2012) 90:3186–99. 10.2527/jas.2011-4342 22585808

[65] Martínez-Miró S Tecles F Ramón M Escribano D Hernández F Madrid J. Causes, consequences and biomarkers of stress in swine: an update. BMC Vet Res. (2016) 12:171. 10.1186/s12917-016-0791-8 27543093 PMC4992232

[66] Lawrence AB Petherick JC McLean KA Deans LA Chirnside J Vaughan A. The effect of environment on behaviour, plasma cortisol and prolactin in parturient sows. Appl Anim Behav Sci. (1994) 39:313–30. 10.1016/0168-1591(94)90165-1

[67] Pedersen LJ Jensen T. Effects of late introduction of sows to two farrowing environments on the progress of farrowing and maternal behavior. J Anim Sci. (2008) 86:2730–7. 10.2527/jas.2007-0749 18469055

[68] Lawrence AB Petherick JC McLean KA Gilbert CL Chapman C Russell JA. Naloxone prevents interruption of parturition and increases plasma oxytocin following environmental disturbance in parturient sows. Physiol Behav. (1992) 52:917–23. 10.1016/0031-9384(92)90371-8 1484848

[69] Yun J Swan KM Vienola K Oliviero C Peltoniemi O Valros A. Effects of prepartum housing environment on abnormal behaviour, the farrowing process, and interactions with circulating oxytocin in sows. App Anim Behav Sci. (2015) 162:20–5. 10.1016/j.applanim.2014.11.006

[70] Yun J Valros A. Benefits of prepartum nest-building behaviour on parturition and lactation in sows – a review. Asian Austr J Anim Sci. (2015) 28:1519. 10.5713/ajas.15.0174 26333669 PMC4647089

[71] Jarvis S Lawrence AB McLean KA Deans L Chirnside J Calvert SK. The effect of environment on behavioural activity, ACTH. β-endorphin and cortisol in pre-farrowing gilts. Anim Sci. (1997) 65:465–72. 10.1017/S1357729800008663

[72] Jarvis S Lawrence AB McLean KA Chirnside J Deans L Calvert SK. The effect of environment on plasma cortisol and b-endorphin in the parturient pig and the involvement of endogenous opioids. Anim Reprod Sci. (1998) 52:139–51. 10.1016/S0378-4320(98)00090-6 9776487

[73] Jarvis S Van der Vegt BJ Lawrence AB McLean KA Deans LA Chirnside J. The effect of parity and environmental restriction on behavioural and physiological responses of pre-parturient pigs. Appl Anim Behav Sci. (2001) 71:203–16. 10.1016/S0168-1591(00)00183-0 11230901

[74] Thodberg K Jensen KH Herskin MS. Nursing behaviour, postpartum activity and reactivity in sows: effects of farrowing environment, previous experience and temperament. Appl Anim Behav Sci. (2002) 77:53–76. 10.1016/S0168-1591(02)00023-0

[75] Jarvis S Calvert SK Stevenson JS Leeuwen N Lawrence AB. Pituitary–adrenal activation in pre-parturient pigs (Sus scrofa) is associated with behavioural restriction due to lack of space rather than nesting substrate. Anim Welf. (2002) 11:371–84.

[76] Yun J Swan KM Vienola K Farmer C Oliviero C Peltoniemi O. Nest-building in sows. Effects of farrowing housing on hormonal modulation of maternal characteristics. App Anim Behav Sci. (2013) 148:77–84. 10.1016/j.applanim.2013.07.010

[77] Stangel G Jensen P. Behaviour of semi-naturally kept sows and piglets (except suckling) during 10 days postpartum. Appl Anim Behav Sci. (1991) 31:211–27. 10.1016/0168-1591(91)90006-J

[78] Jensen P Redbo I. Behaviour during nest leaving in free-ranging domestic pigs. Appl Anim Behav Sci. (1987) 18:355–62. 10.1016/0168-1591(87)90229-2

[79] Berensmann I Klein S Reese S Erhard M Patzkéwitsch D. A comparison of different farrowing systems. Part. 1: Effects on the activity of the sow. Tierarztl Prax Ausg G Grosstiere Nutzt. (2018) 46:291–7. 10.15653/TPG-180491 30340238

[80] Illmann G Hammerschmidt K Špinka M Tallet C. Calling by domestic piglets during simulated crushing and isolation: a signal of need? PLoS ONE. (2013) 8:e83529. 10.1371/journal.pone.0083529 24349527 PMC3862757

[81] Marchant JN Broom DM Corning S. The influence of sow behaviour on piglet mortality due to crushing in an open farrowing system. Anim Sci. (2001) 72:19–28. 10.1017/S135772980005551X

[82] Melišov á M Illmann G Andersen IL Vasdal G Haman J. Can sow pre-lying communication or good piglet condition prevent piglets from getting crushed? Appl Anim Behav Sci. (2011) 134:121–9. 10.1016/j.applanim.2011.06.015

[83] Ocepek M Andersen I. Sow communication with piglets while being active is a good predictor of maternal skills, piglet survival and litter quality in three different breeds of domestic pigs (Sus scrofa domesticus). PLoS ONE. (2018) 13:e0206128. 10.1371/journal.pone.0206128 30427860 PMC6235262

[84] Martin JE Ison SH Baxter EM. The influence of neonatal environment on piglet play behaviour and post-weaning social and cognitive development. Appl Anim Behav Sci. (2015) 163:69–79. 10.1016/j.applanim.2014.11.022

[85] Baxter EM Edwards SA. Optimising sow piglet welfare during farrowing lactation. In: Edwards SA, editor. Understanding the Behaviour and Improving the Welfare of Pigs. Burleigh Dodds (2021). p. 121–76. 10.19103/AS.2020.0081.0434364398

[86] Špinka M Illmann G Algers B Štětková Z. The role of nursing frequency in milk production in domestic pig. J Anim Sci. (1997) 75:1223–8. 10.2527/1997.7551223x 9159268

[87] Špinka M Illmann G Haman J Šimeček P Šilerová J. Milk ejection solicitations and non-nutritive nursings: an honest signaling system of need in domestic pigs? Behav Ecol Sociobiol. (2011) 65:1447–57. 10.1007/s00265-011-1155-9

[88] Arey DS Sancha ES. Behaviour and productivity of sows and piglets in a family system and in farrowing crates. Appl Anim Behav Sci. (1996) 50:135–45. 10.1016/0168-1591(96)01075-1

[89] Su G Lund MS Sorensen D. Selection for litter size at day five to improve litter size at weaning and piglet survivalrate. J Anim Sci. (2007) 85:1385–92. 10.2527/jas.2006-631 17339413

[90] KilBride AL Mendl M Statham P Held S Harris M Cooper S. A cohort study of preweaning piglet mortality and farrowing accommodation on 112 commercial pig farms in England. Prev Vet Med. (2012) 104:281–91. 10.1016/j.prevetmed.2011.11.011 22197175

[91] Petrides JS Mueller GP Kalogeras KT Chrousos GP Gold PW Deuster PA. Exercise-induced activation of the hypothalamic-pituitary-adrenal axis: marked differences in the sensitivity to glucocorticoid suppression. J Clinic Endocrinol Metabol. (1994) 79:377–83. 10.1210/jcem.79.2.8045951 8045951

[92] Cronin GM Barnett JL Hodge FM Smith JA McCallum TH. The welfare of pigs in two farrowing/lactation environments: cortisol responses of sows. Appl Anim Behav Sci. (1991) 32:117–27. 10.1016/S0168-1591(05)80036-X

[93] Biensen NJ von Borell EH Ford SP. Effects of space allocation and temperature on periparturient maternal behaviors, steroid concentrations, and piglet growth rates. J Anim Sci. (1996) 74:2641–8. 10.2527/1996.74112641x 8923178

[94] Jarvis S D'Eath RB Robson SK Lawrence AB. The effect of confinement during lactation on the hypothalamic-pituitary-adrenal axis and behaviour of primiparous sows. Physiol Behav. (2006) 87:345–52. 10.1016/j.physbeh.2005.10.004 16332379

[95] Yin G Liu H Li X Quan D Bao J. Effect of farrowing environment on behaviour and physiology of primiparous sows with 35-day lactation. Int J App Res Vet Sci. (2016) 14:31–7.

[96] Drake A Fraser D Weary DM. Parent–offspring resource allocation in domestic pigs. Behav Ecol Sociobiol. (2008) 62:309–19. 10.1007/s00265-007-0418-y

[97] Wiechers DH Brunner S Herbrandt S Kemper N Fels M. Analysis of hair cortisol as an indicator of chronic stress in pigs in two different farrowing systems. Front Vet Sci. (2021) 8:605078. 10.3389/fvets.2021.605078 33585618 PMC7876061

[98] Broom DM. Animal welfare: concepts and measurement. J Anim Sci. (1991) 69:4167–75. 10.2527/1991.69104167x 1778832

[99] Broom DM Fraser AF. Domestic Animal Behaviour and Welfare. 5th ed. Wallingford: CABI (2015). 10.1079/9781780645391.0000

[100] Damm BI Lisborg L Vestergaard KS Vanicek J. Nest-building, behavioural disturbances and heart rate in farrowing sows kept in crates and Schmid pens. Livest Prod Sci. (2003) 80:175–87. 10.1016/S0301-6226(02)00186-0

[101] Hotzel MJ Machado Filho LCP Dalla Costa OA. Behaviour of pre-parturient sows housed in intensive outdoor or indoor systems. Pesq Agropec Brasil. (2005) 40:169–74. 10.1590/S0100-204X200500020001030466662

[102] Rosvold EM Kielland C Ocepek M Framstad T Fredriksen B Andersen-Ranberg I. Management routines influencing piglet survival in loose-housed sow herds. Livest Sci. (2017) 196:1–6. 10.1016/j.livsci.2016.12.001

[103] Ask B Dahl J Nielsen MB Moustsen V. Comment on: neonatal piglet traits of importance for survival in crates and indoor pens. J Anim Sci. (2012) 90:2879–81. 10.2527/jas.2011-4998 22585827

[104] Damm BI Pedersen LJ Heiskqnen T Nielsen NP. Long-stemmed straw as an additional nesting material in modified schmid pens in a commercial breeding unit: effects on sow behaviour, and on piglet mortality and growth. Appl Anim Behav Sci. (2005) 92:45–60. 10.1016/j.applanim.2004.10.013

[105] Van de Weerd H Ison S. Providing effective environmental enrichment to pigs: how far have we come. Animals. (2019) 9:254. 10.3390/ani9050254 31117191 PMC6562439

[106] Romeo RD Tang AC Sullivan RM. Early life experiences: enduring behavioral, neurological and endocrinological consequences. In: Pfaff DW, Arnorld AP, Etgen AM, Fahrbach SE, Rubin RT. Hormones, Brain and Behavior. Academic Press (2009). p. 1975–2006. 10.1016/B978-008088783-8.00062-0

[107] Langenhof MR Komdeur J. Why and how the early-life environment affects development of coping behaviours. Behav Ecol Sociobiol. (2018) 72:1–32. 10.1007/s00265-018-2452-3 29449757 PMC5805793

[108] Nordquist RE Meijer E van der Staay FJ Arndt SS. Pigs as model species to investigate effects of early life events on later behavioral and neurological functions. In: Animal Models for the Study of Human Disease. Copenhagen: SEGES Danish Pig Research Centre, Academic Press. (2017). 10.1016/B978-0-12-809468-6.00039-5

[109] Bolhuis JE Schouten WGP Schrama JW Wiegant VM. Effects of rearing and housing environment on behaviour and performance of pigs with different coping characteristics. Appl Anim Behav Sci. (2006) 101:68–85. 10.1016/j.applanim.2006.01.001

[110] van Dixhoorn ID Reimert I Middelkoop J Bolhuis JE Wisselink HJ Koerkamp PWG. Enriched housing reduces disease susceptibility to co-infection with porcine reproductive and respiratory virus (PRRSV) and *Actinobacillus pleuropneumoniae* (*A. pleuropneumoniae*) in young pigs. PLoS ONE. (2016) 11:e0161832. 10.1371/journal.pone.0161832 27606818 PMC5015855

[111] Oostindjer M van den Brand H Kemp B Bolhuis JE. Effects of environmental enrichment and loose housing of lactating sows on piglet behaviour before and after weaning. Appl Anim Behav Sci. (2011) 134:31–41. 10.1016/j.applanim.2011.06.011 20622185

[112] Guy JH Cain PJ Seddon YM Baxter EM Edwards SA. Economic evaluation of high welfare indoor farrowing systems for pigs. Anim Welf. (2012) 21:19–24. 10.7120/096272812X1334590567352022436159

[113] Arellano PE Pijoan C Jacobson LD Algers B. Stereotyped behaviour, social interactions and suckling pattern of pigs housed in groups or in single crates. Appl Anim Behav Sci. (1992) 35:157–66. 10.1016/0168-1591(92)90006-W

[114] Baxter EM Lawrence AB Edwards SA. Alternative farrowing accommodation: welfare and economic aspects of existing farrowing and lactation systems for pigs. Animal. (2012) 6:96–117. 10.1017/S1751731111001224 22436159

